# E2 superfamily of ubiquitin-conjugating enzymes: constitutively active or activated through phosphorylation in the catalytic cleft

**DOI:** 10.1038/srep14849

**Published:** 2015-10-14

**Authors:** Ilaria Valimberti, Matteo Tiberti, Matteo Lambrughi, Boris Sarcevic, Elena Papaleo

**Affiliations:** 1 Department of Biotechnology and Biosciences, University of Milano-Bicocca, Piazza della Scienza 2, 20126, Milan (Italy); 2 Cell Cycle and Cancer Unit, St. Vincent’s Institute of Medical Research and The Department of Medicine, St. Vincent’s Hospital, The University of Melbourne, Fitzroy, Melbourne, Victoria 3065, Australia

## Abstract

Protein phosphorylation is a modification that offers a dynamic and reversible mechanism to regulate the majority of cellular processes. Numerous diseases are associated with aberrant regulation of phosphorylation-induced switches. Phosphorylation is emerging as a mechanism to modulate ubiquitination by regulating key enzymes in this pathway. The molecular mechanisms underpinning how phosphorylation regulates ubiquitinating enzymes, however, are elusive. Here, we show the high conservation of a functional site in E2 ubiquitin-conjugating enzymes. In catalytically active E2s, this site contains aspartate or a phosphorylatable serine and we refer to it as the conserved E2 serine/aspartate (CES/D) site. Molecular simulations of substrate-bound and -unbound forms of wild type, mutant and phosphorylated E2s, provide atomistic insight into the role of the CES/D residue for optimal E2 activity. Both the size and charge of the side group at the site play a central role in aligning the substrate lysine toward E2 catalytic cysteine to control ubiquitination efficiency. The CES/D site contributes to the fingerprint of the E2 superfamily. We propose that E2 enzymes can be divided into constitutively active or regulated families. E2s characterized by an aspartate at the CES/D site signify constitutively active E2s, whereas those containing a serine can be regulated by phosphorylation.

Phosphorylation is a major mechanism of post-translational regulatory modification, influencing almost all cellular functions^1^,^2^,^3^. It has been estimated that more than 40% of proteins are phosphorylated *in vivo*^4^. Phosphorylation offers a dynamic mechanism to regulate protein function due to its fast kinetics and reversibility^5^,^6^,^7^. Addition of a phosphate group to serine, threonine or tyrosine residues can induce large conformational modifications in proteins^8^,^9^,^10^,^11^,^12^,^13^,^14^,^15^,^16^,^17^,^18^. This is driven by electrostatic perturbation or steric hindrance, which can affect the protein energy landscape, intermolecular protein-ligand interactions and enzymatic activity^18^,^19^,^20^,^21^,^22^. In addition to the critical role of phosphorylation in normal cellular function, aberrant regulation of phosphorylation-induced electrostatic switches is involved in numerous pathologies, such as cancers^23^,^24^,^25^. High-throughput mass spectrometric studies have identified thousands of post-translational phosphorylation sites on different proteins^26^,^27^. However, precisely defining the role of these modifications on protein structure and dynamics at a molecular and atomic-level is still experimentally challenging.

Cyclin-dependent kinases (Cdks) are key enzymes involved in promoting cell cycle progression in eukaryotic cells^28^. Over the last decade, several studies demonstrated that Cdks can regulate enzymes of the ubiquitination cascade^29^,^30^,^31^,^32^,^33^,^34^,^35^,^36^,^37^,^38^,^39^. The ubiquitination pathway consists of three classes of enzymes, which catalyze the attachment of the protein Ubiquitin (Ub) to various substrates (Fig. 1)^40^. In this enzymatic pathway the Ub C-terminus is first activated by an Ub-activating enzyme (E1) in an ATP-dependent reaction. The activated Ub is subsequently transferred to a conserved cysteine to one of the distinct Ub-conjugating enzymes (E2s), to generate a thioester bond. The E2s then catalyze the transfer of Ub to a substrate lysine to form an isopeptide bond, in conjunction with an Ub-protein ligase (E3), which is also important for substrate selection^41^. Since Ub contains seven lysines, in many cases certain E2/E3 pairs catalyze further rounds of Ub attachment to generate substrates with poly-Ub chains^42^,^43^,^44^.

E2 Ub-conjugating enzymes are critical in this cascade, as they regulate both the topology of the poly-Ub chains and the processivity of the polyubiquitination reaction^45^,^46^,^47^. The human genome encodes more than 40 E2s^45^,^46^,^47^, which catalyze attachment of Ub to different lysines on protein substrate or Ub, during mono- or poly-ubiquitination. This versatility allows for a multitude of distinct ubiquitination events and the generation of diverse protein-Ub structures^42^,^43^,^44^. The E2 superfamily has been divided in 17 families by comprehensive phylogenetic analysis^48^. E2s share a ~150-amino acid conserved catalytic core domain, which is the minimal sufficient unit for enzymatic activity (Fig. 1). This catalytic core domain is known as the Ubiquitin Conjugation (UBC) domain and contains the catalytic cysteine, which forms a thioester bond with Ub^45^,^46^,^47^. E2s recently gained attention as targets to design inhibitory molecules^49^,^50^,^51^,^52^,^53^ since their aberrant regulation is associated with cancer or neurodegenerative diseases^54^,^55^,^56^.

Although several high-resolution three-dimensional (3D) structures of E2 and E3 enzymes have yielded many insights into the mechanisms of Ub or Ubiquitin-like (Ubl) attachment to substrates^41^, many of the fundamental mechanisms of Ub attachment remain to be elucidated. Studies with the small Ubl modifier SUMO and its human cognate E2, Ube2I, identified a triad of conserved residues in Ube2I, which are crucial in activating the substrate acceptor lysine to accept SUMO^57^,^58^. Therefore, Asn85 of the His-Pro-Asn (HPN) motif, Tyr87 and Asp127 play important roles in lowering the substrate lysine pKa to allow its nucleophilic attack of the Ube2I ~ SUMO thioester bond. Several studies have demonstrated the importance of these residues for the catalytic function of different E2s, especially the HPN motif^59^,^60^,^61^,^62^,^63^,^64^. Asp127 plays an important role in the catalytic cleft of Ube2I^57^, a role which is likely to be generally important in E2s. For example, Ser139 in the yeast E2, Cdc34, represents the residue homologous to Ube2I Asp127. Mutation of Cdc34 Ser139 to Asp (Cdc34 S139D), abrogated the ability of Cdc34 to generate lysine 48-linked poly-Ub chains but did not affect the ability of this enzyme to attach Ub to protein substrate^62^. Therefore, mutation of serine 139 from serine to aspartate, converted Cdc34 from a poly-ubiquitinating to a mono-ubiquitinating enzyme^62^. In other E2s, such as *Saccharomyces cerevisiae* Rad6 and its human ortholog hHR6A (Ube2A), the site homologues to Ube2I Asp127 is a serine (Ser120, Fig. 1), whose phosphorylation enhances E2 catalytic activity^29^,^34^. The important regulatory nature of this site is further exemplified by mutational studies, demonstrating that the activity of hHR6A can be upregulated or downregulated, depending on the amino acid in this position^29^,^34^. Hence mutations of hHR6A Ser120 to alanine (S120A) or threonine (S120T) abolished hHR6A Ub-conjugating activity, whereas S120E mutant variant displayed 35% of the activity of the wild type hHR6A. Conversely, a S120D mutation increased the activity of this enzyme three-fold, compared to the wild-type hHR6A, similar to the four-fold increase observed when hHR6A Ser120 is phosphorylated (pS120). Hereafter, we refer to this site as the conserved E2 Ser/Asp site (CES/D).

In this study we investigate this site in the whole E2 superfamily by phylogenetic analysis and more than 60 atomistic MD simulations, which are a powerful approach to investigate the role of phosphorylation in atomic detail^9^,^12^,^15^,^16^,^17^,^18^,^20^,^65^,^66^,^67^,^68^,^69^,^70^,^71^,^72^. We investigate how mutation or phosphorylation of the site affects the dynamics, structure and function of free E2s and E2s in complex with the target substrate. Our studies show that E2 families that are catalytically active conserve a negatively charged residue (Asp) or serine (Ser). Phosphorylation of E2s with a serine at this site, by kinases such Cdks, increases E2 activity^29^,^34^. Our data also indicates that a delicate balance between the size and charge of the CES/D site regulates alignment of the substrate lysine toward the E2 catalytic cysteine to control E2 catalytic activity.

## Results and Discussion

### A conserved E2 Ser/Asp (CES/D) site in catalytically active E2 members is characterized by a constitutive or regulated negatively charged residue

**Figure 2:**
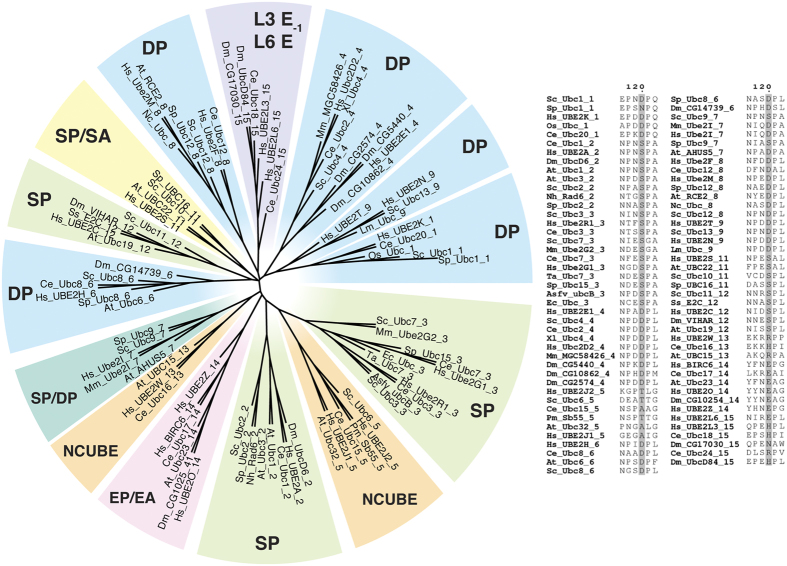
Phylogenetic tree of E2 families with the consensus patterns for the positions corresponding to the CES/D site. In the panel on the right, we reported a portion of the sequence alignment reported in Supplementary Fig. S1 to show the residues in the immediate proximity of the CES/D site. Each sequence is labelled as ‘Organism_E2_Family’ so for example *S. cerevisiae* Ubc2 (Rad6) from family 2 is labelled as ‘Sc_Ubc2_2’. The phylogenetic tree is coloured according to the conserved motif at the CES/D site within each family using light blue, light green, marine, yellow, pink and violet colours for DP, SP, SP/DP, SP/SA, EP/EA and E sequence motifs, respectively. The NCUBE families are highlighted in orange.

In the E2 superfamily, the site, which corresponds to hHR6A Ser120 (CES/D), generally contains an acidic residue (mostly Asp) or a ‘phosphorylatable’ serine residue (Fig. 2, Supplementary Fig. S1 and Table S1). Conversely, catalytically inactive members of the E2 superfamily, which retain the conserved E2 fold but lack a catalytic cysteine and are not involved in conjugation of Ub or Ub-like (Ubl) modifiers, such as the UEVs, do not show a strictly conserved residue at the same site (Supplementary Fig. S2). The only exceptions that we observed are families 5 and 13, which we included in the phylogenetic analysis since most of the members show a conserved cysteine residue at the catalytic site. Family 5 E2s are named Noncanonical Ubiquitin Conjugating Enzymes (NCUBE) as they lack the conserved HPN motif and have structural reorganizations in the region surrounding the catalytic cleft^73^ making difficult a structural alignment with the canonical E2s. Family 13 E2 enzymes do not show a conserved HPN motif in all the members. Moreover a non-canonical Ubl-conjugating activity has been reported for the human member of family 13, i.e., UBE2W^74^,^75^,^76^, so that we labeled this group as NCUBE E2s too. In addition, we should mention that Ube2D4 and Ube2U of family 4 do not feature a canonical Ubl-conjugating activity^44^. Ube2U does not conserve an acid or phosphorylatable residue at the CES/D site, whereas most of the members of family 4 feature an Asp residue at the site. In families 14 and 15 the site is occupied by another negatively charged residue, i.e. a glutamate, which in members of family 15 that are Ube2L3-like are in position −1 (labeled as L3 E_−1_ in Fig. 2) with respect to the canonical CES/D site. To our knowledge, these two families are not extensively characterized, and thus additional studies will be needed to clarify the role of the glutamate at the CES/D site. Our analysis strongly supports the notion that the physical-chemical properties of this E2 site is very important for Ub and Ubl-conjugation activity and that the selective pressure for maintenance of a serine or aspartate at this position is missing in the UEVs or in other proteins, which are deprived of Ubl-conjugation activity but still conserve an E2-like fold.

Interestingly, the conserved site is almost invariably accompanied by a proximal C-terminal proline (Fig. 2, Supplementary Fig. S1). Therefore, E2s with a phosphorylatable serine at the conserved site conform to the consensus phosphorylation motif for Cdks^77^ (Fig. 2, Supplementary Fig. S1). S120 is known to be phosphorylated by Cdk kinases in Rad6^34^ and hHR6A from family 2^29^ and our analysis suggests that it might be phosphorylated in other E2 enzymes. Mutational studies of the CES/D site of hHR6A indicate that a negatively charged residue at this site is important for optimal E2 conjugation activity^29^. An aspartate residue at the CES/D site in Ube2I of family 7 is necessary for the ubiquitination of the target substrate in cooperation with the invariant asparagine of the HPN motif and a tyrosine residue^57^.

In summary, we propose that a negatively charged residue at this site, which can be either constitutive or modulated by phosphorylation, is important for optimal E2 catalytic activity. We here defined an additional conserved site for the E2 superfamily, that together with HPN, the catalytic cysteine and the invariant tryptophan^48^ contribute to define the signature of these enzymes.

### A negatively charged residue of a specific size at the E2 CES/D site allows conserved principal motions of the free enzyme in solution

The conserved patterns observed in our phylogenetic analysis prompted us to compare the 3D structures and dynamics of representative E2 enzymes, which feature either an aspartate or a serine at the CES/D site, by multi-replicate atomistic MD simulations in explicit solvent (Supplementary Table S2). In particular, we selected two E2 enzymes containing a constitutive aspartic acidic residue (yeast Ubc1 and human Ube2I) and three enzymes with serine (yeast Rad6, human hHR6A and hHR6B) at the site. Ube2I is a SUMO-conjugating enzyme and thus we included in our study the Ub-conjugating enzyme Ubc1, which also features an aspartate at the CES/D site for a better coverage of the E2 members. Moreover, several Rad6 and hHR6A serine 120 point mutant variants have been experimentally investigated and provide a rich source of information on the role of the CES/D site in E2 catalytic activity^29^,^34^. We also carried out MD simulations of all these mutants and phospho-variants. The information on the simulated systems and the MD replicates are summarized in the Supplementary Table S2.

**Figure 3:**
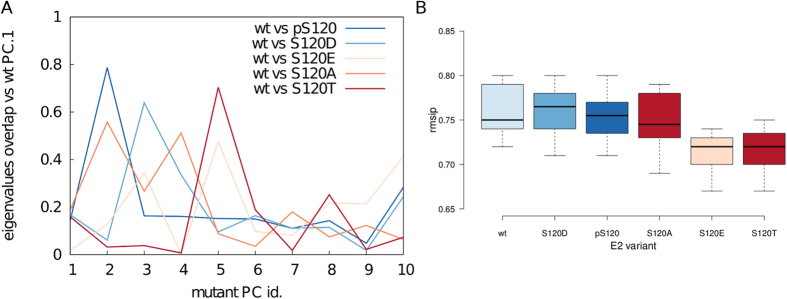
PCA analysis of MD trajectories of wild type, mutant or phosphorylated E2 variants. (**A**) We evaluate if there is a crossing-over of eigenvalues between difference PCs of wild type and mutant E2 variants. The example of the overlap between the eigenvalues of the PC.1 of wild type hHR6A and the eigenvalues of each of the first 10 PCs of hHR6A mutants is shown to illustrate this result. We noticed that an interchange of PCs between wild type and mutant variants and this occurs independently of the effect that the mutation has on E2 Ub-activity. Indeed, it is equally true for active and inactive mutant variants. (**B**) To properly compare our different simulations we estimate the RMSIP between the essential subspaces described by the first 20 PCs of wild-type variants compared each other or compared to S120D, pS120, S120A, S120T and S120E mutant variants respectively. A boxplot analysis of the data has been carried out with *BoxPlotR*^101^. Each dataset is coloured according to the effects on Ub-conjugating activity (different shade of blue for active variants and different shade of orange for partially active or inactive variants). Center lines show the medians, box limit indicates the 25^th^ and 75^th^ percentiles and whiskers extend 1.5 times the interquartile range from the 25^th^ and 75^th^ percentiles.

To compare the dynamic properties and the structural effects induced by different amino acids or phosphorylation at the CES/D site, we carried out a principal component analysis (PCA) of a Cα concatenated trajectory of all the MD simulations^78^ using the same reference subspace and calculating the covariance matrix of corresponding Cα atoms according to the multiple sequence alignment reported in Supplementary Fig. S3. PCA is a technique often employed to compare dynamic properties of different protein variants in an ‘essential’ subspace that retains only the largest amplitude fluctuations^79^,^80^,^81^,^82^. It would be tempting to use the first three eigenvectors (Principal Components, PCs) to provide a 3D representation of the motions described by the different variants. This approach might encounter the risk to emphasize non-genuine differences. Indeed, for different single mutant variants of the same protein or even for proteins with a conserved fold, such as those that we are comparing here, the differences are more likely to be ascribed to changes in the eigenvalues rather than direction of motions. The usage of only the first or the first three PCs can thus encounter the risk of observing differences due to a ‘crossing-over’ of corresponding eigenvalues between difference PCs of two different systems. This is indeed the case in our simulations (Fig. 3A). We estimated the overlap between eigenvalues of the first PCs of the wild type variants and each of the first 10 PCs of the mutant variants, here exemplified in Fig. 3A for the eigenvalues of the first PC of wild type hHR6A which overlaps with higher index PCs (up to the fifth PC) in all the mutants, including either active and inactive variants.

We thus quantified the similarities in protein dynamics of the different variants using the first 20 PCs, which account for more than 80% of the total variance of the system. We calculated the root mean square inner product (RMSIP) as a measure of overlap. In particular, we carried out a boxplot analysis (Fig. 3B) comparing the RMSIP values obtained between different classes. Indeed, we compared the RMSIP values obtained when only wild-type E2 enzymes (hHR6A, hHR6B, UBC1 and UBE2I) are compared to each other with the RMSIP values of wild-type E2s vs S120D, pS120, S120A, S120E and S120T variants, respectively. We can see a generally conserved dynamics with high RMSIP values, i.e. quite overlapping essential subspaces. There are no radical changes, but we can observe a subtle effect due to fully inactive S120T and partially active S120E mutations, which feature slightly lower RMSIP values when compared to the wild type E2s, a signal of potential perturbation induced on native dynamics upon these mutations. S120A, which is also an inactive mutation for Ub-conjugating activity is characterized by a native-like behaviour in the free E2, suggesting that S120A does not affect the dynamics and stability of the free protein and it is more likely to exert an effect directly with respect to the substrate binding.

In summary, a PCA analysis of our MD trajectories shows that mutations at the CES/D site that abolish or impair (S120E and S120T) the Ub-conjugation activity induce perturbations in E2 native dynamics, whereas the other variants do not feature any remarkable effects.

### Phosphorylation of CES/D site increases the solvent accessibility of the catalytic cysteine

**Figure 4:**
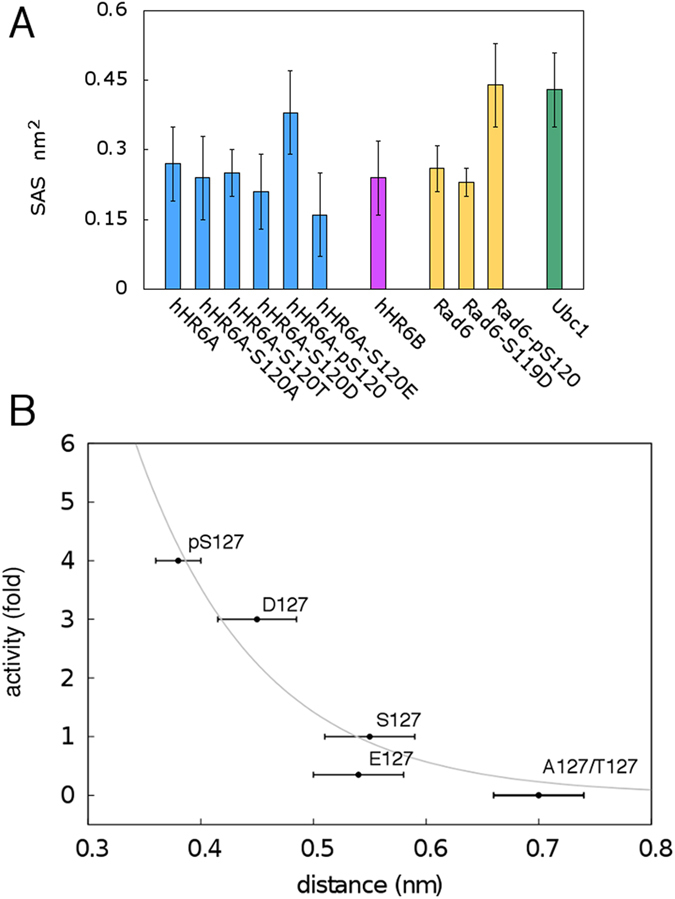
(**A**) Average Solvent Accessible Surface (SAS) of the side chain of the E2 catalytic cysteine in the simulations of the wild type, mutant or phosphorylated E2 variants. The SAS (nm^2^) of the side-chain atoms of the catalytic cysteine is reported as an average value over four replicates of each variants and the corresponding bars indicate the associated standard deviations. hHR6A, hHR6B, Rad6 and Ubc1 variants are shown in blue, magenta, yellow and dark green, respectively. (**B**) Correlation between the Ub-conjugating activity and the distance between the catalytic cysteine of the E2 donor and the side chain of the substrate acceptor lysine in wild type, mutant and phosphorylated Ube2I-RanGAP1 complexes. The experimental data are taken from^29^. Each point represents the average distance calculated over four replicates of each variant and the corresponding bars indicate the associated standard deviations. The plot has been derived upon exponential fitting of the data according to f(x) = e^−(x*c)^ * b with fit parameters b and c set equal to 136.28 and 9.12, which provide a correlation of 0.98.

To further unveil the effects induced upon phosphorylation or mutation in the active site, we calculated the solvent accessible surface (SAS) of the catalytic cysteine in our simulations. The phospho-hHR6A and -Rad6 variants present a higher average solvent accessibility of the cysteine than the non-phosphorylated or S120D counterparts (hHR6A and Rad6) (Fig. 4A). The solvent accessibility of the phospho-variants is comparable to accessibility of Ubc1, in which an aspartate residue occupies the CES/D site (Fig. 4A). Ube2I contains an insertion in proximity of the active site, which corresponds structurally to a loop that protrudes from the catalytic cleft and resembles the acidic loop of Cdc34-like E2 enzymes^83^. Opening/closing motions of this loop may partially occlude the catalytic cysteine, as observed for Cdc34^83^. Thus we did not include Ube2I in the SAS analysis since we are interested in assessing if mutations within the catalytic cleft correlate with accessibility of the catalytic cysteine and E2 activity. Mutations to alanine or threonine do not affect the accessibility of the catalytic cleft overall. Conversely, the glutamate mutation introduces a longer side chain in the catalytic cleft compared to either pS120 or the S120D mutation, partially occluding the catalytic cysteine.

### A subtle balance between charge and size at the CES/D site is required for optimal Ub-conjugation activity

Mutations of the conserved CES/D site of hHR6A, Rad6 or Ube2I do not remarkably affect the Ub/Ubl-charging step of the E2, i.e., the step in which the E1 enzyme transfers the Ub/Ubl to the cognate E2^29^,^57^. Rather, the mutations are mainly impacting on the E2 Ub/Ubl-conjugation activity^29^,^34^,^57^, i.e. the step in which the Ub/Ubl-loaded E2 enzyme, alone or together with the cognate E3, transfers the Ub/Ubl to the lysine of the target substrate. Thus, we investigate if different side chains at the CES/D site influence the orientation of the substrate lysine side chain by MD simulations of Ube2I-RanGP1 wild type (Asp127) and mutant complexes (D127S, D127E, D127T, D127A and 127phospho-S). We selected this complex for our simulations since experimental structures of hHR6A in complex with its target substrate are not available and cannot be modelled by homology. MD simulations are known to encounter the risk of artefacts when they are initialized with a modelled structure rather than an experimental one^84^, especially if sequence identity between targets and templates is low. Moreover, we limited our MD study to describe the local changes in the residues that are in the proximity of either the catalytic cysteine, the CES/D site or the acceptor lysine of the substrate.

We first monitored the distances between the substrate lysine and either CES/D side chain or the catalytic cysteine. We observed that the catalytically active or partially active variants (D127, D127S, pS127 and D127E) allow for interactions between the residue at the CES/D site and the substrate lysine at distances below 0.48 nm, with the phospho-variant exhibiting the minimal distance of ~0.38 nm. The interaction is not observed when alanine or threonine occupies the CES/D (distances higher than 0.7 nm). A similar trend is observed when we calculated the distances between the side chains of the substrate lysine and the E2 catalytic cysteine. The distances between the catalytic cysteine and target lysine residues in our simulated E2-RanGAP1 complex, correlate with an exponential behaviour to the experimentally observed Ub-conjugation activity of hHR6A on its substrate histone H2A^29^ (Fig. 4B). The phospho-variant has the minimal distance between the two residues at values lower than 0.35, thus very close to the optimal distance for Ub/Ubl transfer to the target lysine^85^. Conversely, the interaction between the lysine and the catalytic cysteine in the inactive variants (D127A and D127T) is sub-optimal, with the distance increasing up than 0.7 nm (Fig. 4B). The optimal distance for Ub/Ubl-transfer between the catalytic cysteine and the acceptor lysine of the substrate is expected to be around 0.3–0.35 nm^85^. Therefore, even minor perturbations can cause detrimental effects on E2 Ub/Ubl-conjugation activity, where even small changes in the distance between the catalytic cysteine and the target lysine are correlated to a marked drop in activity for the D127A and D127T mutants.

**Figure 5:**
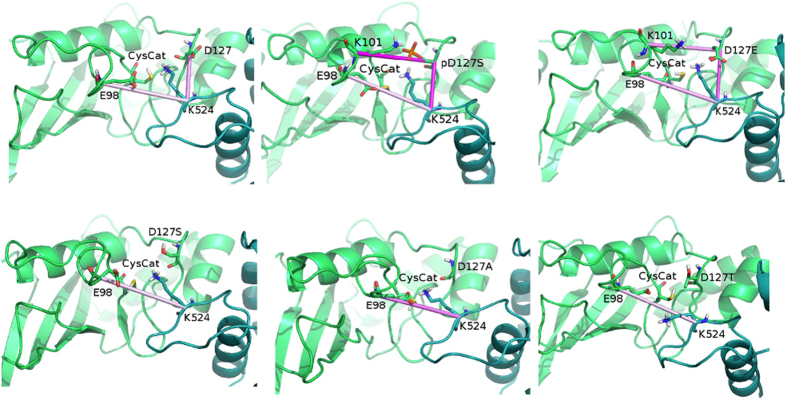
Network of electrostatic interactions between the substrate lysine and the residues surrounding the catalytic cleft of the E2 enzyme in MD simulations of wild type, mutant and phosphorylated Ube2I-RanGAP1 complexes. RanGAP1 and the E2 enzyme are shown as green and blue marine cartoons, respectively. Pink lines represent salt bridges with different shades of colors according to their persistence in the MD simulations.

We thus speculate that the Ub transfer from the E2 catalytic cysteine to the target lysine is facilitated by the closer interaction between the E2 ~ Ub thioester and the acceptor lysine. Moreover, the negatively charged group of the phospho-Ser can efficiently maintain the target lysine side chain in a conformation suitable for interaction with the catalytic cysteine, due to the electrostatic nature of the interaction between the residue at the E2 CES/D site and the target lysine of the substrate. Indeed, an ion pair and hydrogen bonds are observed between the side chains of the two residues in all the simulation frames of Ube2I-RanGAP1_p127S_ simulations (Supplementary Table S3, Fig. 5). The variant with a serine at this site can still interact with the lysine by hydrogen bonds but the interaction is weaker and observed in 10% of the Ube2I-RanGAP1_D127S_ simulations (Supplementary Table S3, Fig. 5).

The mutations to alanine or threonine cannot provide an interaction with the target lysine and causes a departure of the lysine side chain from the E2 catalytic cleft (Supplementary Table S3, Fig. 5 and Supplementary Fig. S4). We could have expected that the threonine, being a polar residue, could still interact with the target lysine. On the contrary, we observed that the –CH_3_ group of threonine side chain is placed in a way that does not allow for interaction with the substrate lysine (Supplementary Fig. S4).

Moreover, we observed that the residue at the CES/D site is involved in a larger network of electrostatic interactions that contribute to the positioning of the substrate lysine within the E2 catalytic cleft (Fig. 5) in Ube2I-RanGAP1, Ube2I-RanGAP1_pS127_ and Ube2I-RanGAP1_D127E_. The network includes Glu98 in all the three systems and also Lys101 in RanGAP1_pS127_ and Ube2I-RanGAP1_D127E_. The substrate lysine is indeed bridging both the negatively charged residue at position 127 and the Glu98. In the inactive mutants the interaction of the lysine with the CES/D site is lost and the interaction with Glu98 is not sufficient to provide a proper orientation of the substrate lysine within the E2 catalytic cleft (Fig. 5 and S4). Indeed, the Glu98 attracts and positions the side chain of the substrate lysine outside the catalytic pocket.

The presence of a glutamate residue at position 127 allows for a tight interaction with the lysine of the substrate (Supplementary Table S3) but it impacts the lysine-cysteine distance, which is higher than 0.54 nm. The longer side chain of the glutamate residue with respect to an aspartate induces, in some of the simulated frames, a conformation of the lysine not optimal for interaction with the E2 catalytic cysteine, by attracting the lysine side chain away from the cysteine and outside the E2 catalytic cleft (Fig. 5 and S4). We observe that the D127E mutation results in a simulated conformational ensemble of the E2-substrate complex in which only a minor population of the substrate lysine is in a conformation competent for Ub/Ubl-conjugation. The majority of the conformations are not optimal for the activity of the enzyme, explaining the 75% drop in Ub-conjugating activity observed *in vitro*^29^. The active variants, especially pS127, provide a suitable balance between side chain charge and size to maintain the substrate lysine closer to the catalytic site with the lysine side chain bridging the negatively charged residue at position 127 and the Glu98 (Fig. 5).

## Discussion

Studies on Ube2I, which is the E2 enzyme specific for SUMO Ub-like modifier, showed that mutations at Asn85, Tyr87, and Asp127 result in defects in SUMO-conjugating activity to the substrate lysine, as severe as those achieved upon mutation of the catalytic cysteine^57^. Asp127 corresponds to Ser120 of hHR6A, which is phosphorylated by CDKs^29^. Phosphorylation or the mutation of Ser120 to aspartate can substantially increase the ubiquitin-conjugating activity of this enzyme^29^. The catalytic defects observed for the Ube2I mutants have been ascribed to the inability of the mutated residues to coordinate the substrate lysine side chain within the E2 active site. This has been suggested by the observation that the lysine side chain is poorly ordered in the crystallographic structures of the Ube2I mutant variants in complex with the substrate. In addition, these residues decrease the lysine pKa in a range where catalysis can occur under physiological pH conditions, uncovering a dual role for this triad of residues^57^.

Despite the central role that site 120 has in E2 enzymes, we have very limited knowledge of its role in E2 structure, dynamics and function, especially at the atomic level.

Progresses in genome sequencing and annotation of E2 enzymes, allowed the classification of E2 enzymes in 17 families of orthologs^48^. We added new E2 enzymes from diverse organisms to the original pool of E2 sequences and focused on members of E2 families for which a catalytic Ub/Ubl-conjugating activity has been proved. Our analysis shows that the position corresponding to Ser120 of hHR6A is highly conserved in all the catalytically active E2 enzymes, allowing us to classify this position as a new CES/D functional site in E2s. At this position, generally a serine or negatively charged aspartic residue has been selected by evolution. The serine can be phosphorylated to generate a negatively charged side chain. Our studies demonstrate that not only is the charge of this residue important but also its size. Therefore, substitution with the longer glutamic acid side chain results in a less efficient enzyme^29^. Our study suggests this is due to compromised native dynamics and the inability to efficiently coordinate the target lysine of the substrate in the catalytic site. Specifically, we show that when a residue distinct from Asp or the phospho-Ser is located at the CES/D site, the substrate acceptor lysine side chain is more mobile and results in orientations that fail to interact efficiently with the catalytic cysteine. We also observed a correlation between the Ub/Ubl-conjugating activity and the distance between the acceptor lysine side group and the E2 catalytic cysteine. It has been estimated that the optimal distance for Ub/Ubl transfer from the E2 ~ Ub thioester to the substrate acceptor lysine is lower than 0.35 nm^85^. We observed the closest distances with E2s containing a phospho-Ser or an Asp at the CES/D site. Remarkably, even minor changes in the distance between the lysine and the catalytic cysteine cause rather drastic effects in Ub-conjugation activity.

**Figure 6:**
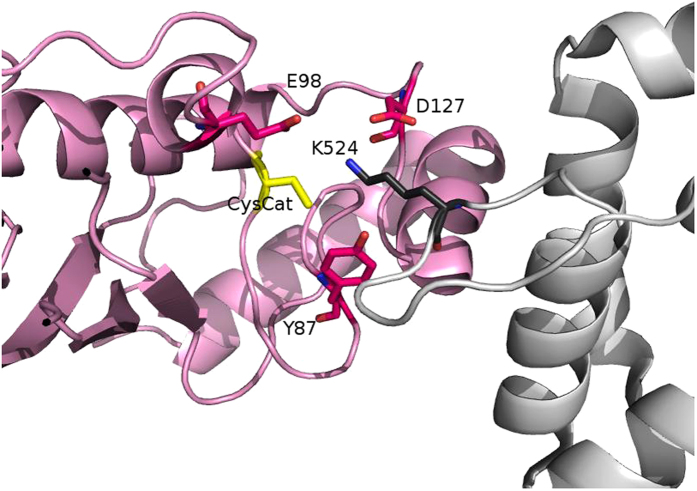
Tyr87, Glu98 and the CES/D residue cooperate in providing the optimal coordination for the acceptor lysine side chain within the catalytic cleft of the E2 enzyme. RanGAP1 and Ube2I are shown as grey and pink cartoons, respectively.

In addition to the importance of the conserved serine/aspartate site, other key residues in the E2 catalytic site can be identified. In particular, Glu98 contributes to a tridentate network of electrostatic interactions, Glu98-Lys_substrate_-CES/D127. Tyr87 is another crucial site for the coordination of the substrate lysine acceptor side chain and cooperates with the CES/D site^57^. The aromatic side chain of this residue can retain the lysine within the E2 catalytic cleft by steric effects (Fig. 6). Indeed, the mutation Y87A is known to reduce the Ubl-conjugating activity in Ube2I, similar to that observed for the D127A mutation^57^. Conversely, a conservative Y87F mutation that retains the properties of the tyrosine side chain does not alter the activity of the enzyme^57^. In agreement with these data, our multiple alignment shows that tyrosine or phenylalanine residues are conserved at the positions homologous to Ube2I Y87 site (Supplementary Fig. S1).

Residues at three sites, Tyr87, Asp or Ser at CES/D site and to a minor extent, Glu98 (Ube 2I numbering) thus cooperate for the optimal coordination of the substrate lysine within the E2 catalytic pocket (Fig. 6). Furthermore, phosphorylation of Ser at the CES/D site can generate a negative charge in this position to increase activity and therefore this site can regulate E2 activity through post-translational modification. The ability to dynamically and reversibly modulate the CES/D site through phosphorylation is likely to have crucial importance for E2 enzymes which need to be more active at a specific times in the cell, such as during cell cycle progression or particular signalling events. Our MD investigation provides insight on the role of the CES/D site at an atomic level. This residue is able to efficiently coordinate the substrate lysine by a subtle balance between its charge and size. The conservation of a phosphorylatable serine or an aspartate in almost all catalytically active E2s suggests that this is a general mechanism for the E2 Ub/Ubl-conjugating enzymes. The CES/D motif in the E2 superfamily characterized in this study, together with the catalytic cysteine, the HPN motif and the invariant Trp, defines a fingerprint of this superfamily of enzymes at the heart of the ubiquitination cascade.

## Methods

### Multiple sequence alignment and phylogenetic analysis

We obtained the unrooted phylogenetic tree using the PHYLIP package^86^ on the basis of a multiple sequence alignment that has been carried out with ClustalW2^87^. The multiple sequence alignment was manually corrected according to information on functional or structural conserved residues and secondary structures, along with a comparison with a structural alignment obtained by DALI and it is reported in Supplementary Fig. S1^88^. The multiple sequence alignment includes the sequences of the UBC domains of several E2 enzymes belonging to each of the 17 E2 families^48^. We discarded E2 enzymes belonging to families 10 and 16 from the analysis since they do not feature a catalytic cysteine and are known to be catalytically inactive^48^,^73^. We also discarded E2 enzymes from family 17 since they do not present a canonical fold of the UBC domain^48^. The sequences corresponding to the UBC domain of each E2 enzymes have been determined by intra-family sequence alignments, since for each family at least one member with known 3D structure is available. Initially, we collected more than 250 E2 sequences and redundant sequences were discarded by DivergentSet program^89^, leading to a selection of 85 sequences in the final alignment (Supplementary Table S1 and Fig. S1). The phylogenetic analysis was carried out using bootstrap with 100 replicates and 12 trials. We employed the Maximum Likelihood method using the Proml program and a Jones-Taylor-Thornton substitution matrix. FigTree software (http://tree.bio.ed.ac.uk/software/figtree) was used for the tree illustration.

### Molecular dynamics simulations

A summary of the starting structures, number of replicates and simulation length used for simulations of wild-type hHR6A, hHR6B, Rad6, Ube2I and Ubc1, along with hHR6A_S120A_, hHR6A_S120T_, hHR6A_S120E_, hHR6A_S120D_, hHR6A_pS120_, Rad6_S120A_, Rad6_S120D_, Rad6_pS120_ is provided in Supplementary Table S1. hHR6A variants were modelled using as template hHR6B. They share 96% sequence identity and do not feature any insertion or deletion in the sequence. The modelling procedure was carried out with Modeller v.9.3^90^ and the final model evaluated using the AIDE software^91^, which classifies them as excellent quality models with predicted root mean square deviation (RMSD) from the native structure, TM-score and LG-score of 0.14 nm, 0.78 and 0.25, respectively. The mutant variants have been selected according to the mutants previously investigated experimentally^29^,^34^. Simulations of Ube2I-RanGAP1 complex have been also carried out for both the wild type (Asp127) and all the mutant variants studied on the free proteins (Ube2I_D127S_-RanGAP1, Ube2I_D127A_-RanGAP1, Ube2I_D127E_-RanGAP1, Ube2I_D127T_-RanGap1 and Ube2I_pS127_-RanGAP1).

MD simulations were performed with Gromacs 4.5^92^ and a modified version of the Gromos 54a7 force field^93^ to account for phospho-residues, as provided by VIENNA-PTM server^94^,^95^. The systems were solvated in a dodecahedral box (minimum distance between protein and box edges: 0.9 nm) of SPC (Simple Point Charge) water molecules, applying periodic boundary conditions. The ionization state of residues was set to be consistent with neutral pH and the tautomeric form of histidine residues was derived using GROMACS tools and confirmed by visual inspection. The system was equilibrated according a protocol previously applied to other E2 enzymes^12^,^96^. We collected overall 64 MD simulations with two or four replicates for each system, collecting in total more than nine μs of MD sampling (Supplementary Table S2). The use of multiple MD replicates of the same system can help to identify recurring features and to avoid simulations artifacts^97^. Simulations were carried out in the isothermal-isobaric ensemble at 300 K at 1 atm using an external Berendsen bath with thermal and pressure coupling of 0.1 and 1 ps, respectively. The LINCS algorithm was used to constraint the heavy atom bonds to use a two fs time-step. Long-range electrostatic interactions were calculated using the Particle-mesh Ewald (PME) summation scheme. Van der Waals and Coulomb interactions were truncated at 0.9 nm.

We discarded the first ten ns of MD simulations from analysis, since they are required to reach stable main-chain root mean square values in all the MD replicates (Supplementary Figs S5–S7). For each system, the equilibrated portions of each replicate were joined together in a macro-trajectory, which is representative of the different direction of sampling around the starting structure of that system.

### Principal Component Analysis (PCA)

PCA of MD trajectories reveals large amplitude concerted motions in the MD ensemble through the eigenvectors of the mass-weighted covariance matrix of the atomic positional fluctuations^78^. We thus carried out PCA using either Cα or all protein atoms on the macro-trajectories of the wild type and mutant variants of E2 enzymes collected in our study using as a guide the alignment reported in Supplementary Fig. S3. We indeed used the same reference subspace to better compare them. The similarity between two eigenvectors, each calculated from a different MD simulation, was evaluated by computing the inner product between them. We have used this method to systematically compare the most relevant eigenvectors of different protein variants to verify where a interchange between eigenvalues of different principal components (PCs) was occurring. We could observe a crossing-over of eigenvalues in the first 10 PCs of different systems. We thus compared pairwise the first 20 PCs of each system using as a measure of overlap the Root Mean Square Inner Product (RMSIP)^98^,^99^.

The cosine content of the principal components (eigenvectors) of the covariance matrix of atomic positional fluctuations is an index that can range between 0 (no cosine) and 1 (perfect cosine). It has been demonstrated that insufficient sampling lead to high cosine content values in MD simulations, representative of random motions^100^. We thus calculated the cosine content of the first 20 PCs of each MD replicate, achieving values always lower than 0.30.

## Additional Information

**How to cite this article**: Valimberti, I. *et al.* E2 superfamily of ubiquitin-conjugating enzymes: constitutively active or activated through phosphorylation in the catalytic cleft. *Sci. Rep.* **5**, 14849; doi: 10.1038/srep14849 (2015).

## Supplementary Material

Supplementary Information

## Figures and Tables

**Figure 1:**
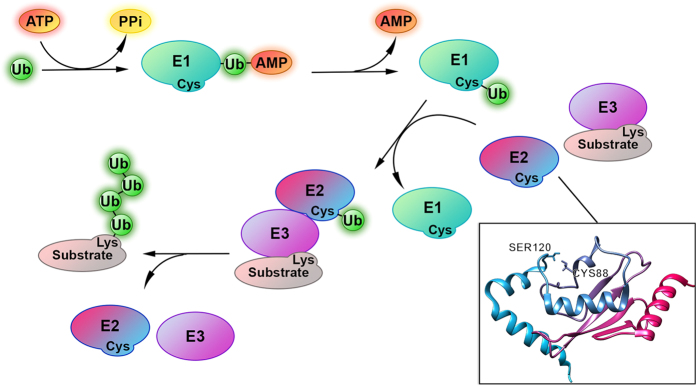
The process of ubiquitination consists of the sequential and cooperative actions of three classes of enzymes that catalyze the attachment of the protein Ubiquitin (Ub, green spheres) to substrate proteins. Ub is first activated by ubiquitin-activating enzyme (E1, green ovals) by an ATP-dependent reaction. The Ub-AMP product remains bound to the E1. Then a cysteine residue attacks the Ub, leading to an E1 ~ Ub thioester intermediate. Subsequently the Ub is transferred to a conserved cysteine in the active site of an ubiquitin-conjugating enzyme (E2, hot pink-azure ovals). The E2s catalyze the transfer of Ub to a lysine on the substrate (pink-grey ovals) to form an isopeptide bond, together with an ubiquitin ligase (E3, purple ovals). Alternatively, E2s can transfer Ub to a catalytic cysteine on HECT family E3s, which then transfer Ub onto substrate to form an isopeptide bond. E3s are important for recognition and selection of the substrate. Certain E2/E3 pairs catalyze several rounds of Ub attachment to generate substrates with poly-Ub chains. Substrate can be mono or poly-ubiquitinated and these modifications mark the substrate for different destiny and functions. In the bottom-right panel the structure of Human Ubiquitin-conjugating enzyme E2 B (PDB ID 1JAS, hHR6B) is showed as cartoon. The catalytic cysteine (Cys88) present in the Ubiquitin Conjugation (UBC) domain of E2, that forms thioester bond with Ub, and the serine (Ser120) in the conserved E2 Ser/Asp site (CES/D), whose phosphorylation enhances E2 catalytic activity, are highlighted as sticks.
