# Supplementary material for: E2 superfamily of ubiquitin-conjugating enzymes: constitutively active or activated through phosphorylation in the catalytic cleft
Source: Sci Rep. 2015 Oct 14;5:14849. doi: 10.1038/srep14849 (PMC4604453; doi:10.1038/srep14849)

Supplementary Information

E2 superfamily of ubiquitin-conjugating enzymes: constitutively active or activated through phosphorylation in the catalytic cleft

Ilaria Valimberti[1], Matteo Tiberti[1], Matteo Lambrughi[1], Boris Sarcevic[2], Elena Papaleo[1]*‡

[1]Department of Biotechnology and Biosciences, University of Milano-Bicocca, Piazza della Scienza 2, 20126, Milan (Italy). [2]Cell Cycle and Cancer Unit, St. Vincent's Institute of Medical Research and The Department of Medicine, St. Vincent's Hospital, The University of Melbourne, Fitzroy, Melbourne, Victoria 3065, Australia

* To whom correspondence should be addressed. Email: elena.papaleo@unimib.it, elena.papaleo.78@gmail.com

‡ Current address: Unit of Statistics, Bioinformatics and Registry, Danish Cancer Society Research Center, Strandboulevarden 49, 2100, Copenhagen, Denmark

Table S1. Information on the E2 sequences used for the multiple sequence alignment in Figure S1 and the phylogenetic analysis reported in Fig1.

Label	Family	Organism	Protein Names	Uniprot ID
Sc_Ubc1_1	1	S.cerevisiae	Ubc1	P21734
Sp_Ubc1_1	1	S.pombe	Ubc1	O74810
Hs_UBE2K_1	1	H.sapiens	UBE2K/E2_25k/HIP2	P61086
Os_Ubc1_1	1	O.sativa	Ubc1	Q8S1Y5
Ce_Ubc20_1	1	C.elegans	Ubc20	Q9TZ69
Ce_Ubc1_2	2	C.elegans	Ubc1	P52478
Hs_UBE2A_2	2	H.sapiens	UBE2A/hHR6A	P49459
Dm_UbcD6_2	2	D.melanogaster	UbcD6	P25153
At_Ubc1_2	2	A.tahaliana	Ubc1	P25865
At_Ubc3_2	2	A.tahaliana	Ubc3	P42746
Sc_Ubc2_2	2	S.cerevisiae	Ubc2/Rad6	P06104
Nh_Rad6_2	2	N.haematococca	Rad6	P78717
Sp_Ubc2	2	S.pombe	Ubc2	P23566
Sc_Ubc3_3	3	S.cerevisiae	Ubc3/Cdc34	P14582
Hs_Ube2R1_3	3	H.sapiens	Ube2R1/Cdc34	P49427
Ce_Ubc3_3	3	C.elegans	Ubc3	Q95XN7
Sc_Ubc7_3	3	S.cerevisiae	Ubc7	Q02159
Mm_Ube2G2_3	3	M.musculus	Ube2G2	P60605
Ce_Ubc7_3	3	C.elegans	Ubc7	P34477
Hs_Ube2G1_3	3	H.sapiens	Ube2G1	P62253
Ta_Ubc7_3	3	T.aestivum	Ubc7	P25868
Sp_Ubc15_3	3	S.pombe	Ubc15	Q9Y818
Asfv_ubcB_3	3	African swine fever virus	ubcB	P25869
Ec_Ubc3_3	3	E.cuniculi	Ubc3	Q8SS54
Hs_UBE2E1_4	4	H.sapiens	UBE2E1/UbcH6	P51965
Sc_Ubc4_4	4	S.cerevisiae	Ubc4	P15731
Ce_Ubc2_4	4	C.elegans	Ubc2/Let70	P35129
Xl_Ubc4_4	4	X.leavis	Ubc4	P62840
Hs_Ubc2D2_4	4	H.sapieins	Ubc2D2/UbcH5B	P62837
Mm_MGC58426_4	4	M.musculus	MGC58426	A2AFH2
Dm_CG5440_4	4	D.melanogaster	CG5440	Q9VQ00
Dm_CG10862_4	4	D.melanogaster	CG10862	Q9I7T6
Dm_CG2574_4	4	D.melanogaster	CG2574	Q9VYN3
Hs_UBE2J2_5	5	H.sapiens	NCUBE2	Q8N2K1
Sc_Ubc6_5	5	S.cerevisiae	Ubc6	P33296
Ce_Ubc15_5	5	C.elegans	Ubc15/ISO A	Q18931
Pm_Sb55_5	5	P.mariana	Sb55	Q65079

At_Ubc32_5	5	A.tahaliana	Ubc32	Q9LSP7
Hs_UBE2J1_5	5	H.sapiens	NCUBE1	Q9Y385
Hs_UBE2H_6	6	H.sapiens	UBE2H	P62256
Ce_Ubc8_6	6	C.elegans	Ubc8	Q9N2W9
At_Ubc6_6	6	A.tahaliana	Ubc6	P42750
Sc_Ubc8_6	6	S.cerevisiae	Ubc8/UbcH2	P28263
Sp_Ubc8_6	6	S.pombe	Ubc8	O74810
Dm_CG14739_6	6	D.melanogaster	CG14739	Q9VGD6
Sc_Ubc9_7	7	S.cerevisiae	Ubc8	P50623
Mm_Ube2I	7	M.musculus	Ube2I	P63280
Hs_Ube2I	7	H.sapiens	Ube2I	P63279
Sp_Ubc9_7	7	S.pombe	Ubc9	P40984
At_AHUS5_7	7	A.tahaliana	AHUS5	Q42551
Hs_Ube2F_8	8	H.sapiens	Ube2F	Q969M7
Ce_Ubc12_8	8	C.elegans	Ubc12	Q9XVK5
Hs_Ube2M_8	8	H.sapiens	Ube2M	P61081
Sp_Ubc12_8	8	S.pombe	Ubc12	O74549
At_RCE2_8	8	A.tahaliana	RCE2	Q9ZU75
Nc_Ubc_8	8	N.crassa	Ubc-like	Q8X018
Sc_Ubc12_8	8	S.cerevisiae	Ubc12	P52491
Hs_UBE2T_9	9	H.sapiens	UBE2T	Q9NPD8
Sc_Ubc13_9	9	S.cerevisiae	Ubc13	P52490
Hs_UBE2N_9	9	H.sapiens	UBE2N	P61088
Lm_Ubc_9	9	L.major	Ubc9	Q9NED4
Hs_UBE2S_11	11	H.sapiens	UBE2S	Q6NXQ4
At_UBC22_11	11	A.tahaliana	UBC22	Q9FF66
Sc_Ubc10_11	11	S.cerevisiae	Ubc10	P29340
Sp_UBC16_11	11	S.pombe	UBC16	Q9P6I1
Sc_Ubc11_12	12	S.cerevisiae	Ubc11	P52492
Ss_E2C_12	12	S.solidissima(clam)	E2C	Q95044
Hs_UBE2C_12	12	H.sapiens	UBE2C	O00762
Dm_VIHAR_12	12	D.melanogaster	VIHAR	Q9VTY6
At_Ubc19_12	12	A.tahaliana	Ubc19	Q9LJZ5
Hs_UBE2W_13	13	H.sapiens	UBE2W	Q96BO2
Ce_Ubc16_13	13	C.elegans	Ubc16	Q9XWF6
At_UBC15_13	13	A.tahaliana	UBC15	P42743
Hs_BIRC6_14	14	H.sapiens	BIRC6	Q9NR09
Ce_Ubc17_14	14	C.elegans	Ubc17	Q11079
At_Ubc23_14	14	A.tahaliana	Ubc23	Q9ZVX1
Hs_UBE2O_14	14	H.sapiens	UBE2O	Q9C0C9

Dm_CG10254_14	14	D.melanogaster	CG10254	Q9XZ32
Hs_UBE2Z_14	14	H.sapiens	UBE2Z	A6NC60
Hs_UBE2L6_15	15	H.sapiens	UBE2L6	O14933
Hs_UBE2L3_15	15	H.sapiens	UBE2L3/UbcH7	P68036
Ce_Ubc18_15	15	C.elegans	Ubc18	Q21633
Dm_CG17030_15	15	D.melanogaster	CG17030	Q9VZ73
Ce_Ubc24_15	15	C.elegans	Ubc24	Q20617
Dm_UbcD84_15	15	D.melanogaster	UbcD84	P52486

Table S2. Summary of our MD simulations of E2 variant and their complexes.

E2 variant	Organism/Source	Catalytic site residues	Number and duration of *replicas*	Starting structure
hHR6A	Human	Cys88<br><br>CES/D site: Ser120	4 replicates x 160 ns each (total duration 0.64 µs)	wild type hHR6A homology model using hHR6B NMR structure (pdb entry 1JAS[1]) as template (sequence identity 96%)
hHR6A-pS120	Human	Cys88<br><br>CES/D site: pSer120	4 replicates x 160 ns each (total duration 0.64 µs)	phospho-Ser120 hHR6A homology model using hHR6B NMR structure (pdb entry 1JAS[1]) as template
hHR6A-S120D	Human	Cys88<br><br>CES/D site: Asp120	4 replicates x 160 ns each (total duration 0.64 µs)	S120D hHR6A homology model using hHR6B NMR structure (pdb entry 1JAS[1]) as template
hHR6A-S120A	Human	Cys88<br><br>CES/D site: Ala120	4 replicates x 160 ns each (total duration 0.64 µs)	S120A hHR6A homology model using hHR6B NMR structure (pdb entry 1JAS[1]) as template
hHR6A-S120T	Human	Cys88<br><br>CES/D site: Thr120	4 replicates x 160 ns each (total duration 0.64 µs)	S120T hHR6A homology model using hHR6B NMR structure (pdb entry 1JAS[1]) as template
hHR6A-S120E	Human	Cys88<br><br>CES/D site: Glu120	4 replicates x 160 ns each (total duration 0.64 µs)	S120E hHR6A homology model using hHR6B NMR structure (pdb entry 1JAS[1]) as template
Rad6	*S.cerevisiae*	Cys87<br><br>CES/D site: Ser119	4 replicates x 160 ns each (total duration 0.64 µs)	X-ray structure of wild-type Rad6 (pdb entry 1AYZ[2])
Rad6-pS120	*S.cerevisiae*	Cys87<br><br>CES/D site: pSer119	4 replicates x 160 ns each (total duration 0.64 µs)	*In-silico* mutation starting from the X-ray structure of wild-type Rad6 (pdb entry 1AYZ[2])
Rad6-S120D	*S.cerevisiae*	Cys87<br><br>CES/D site: Asp119	4 replicates x 160 ns each (total duration 0.64 µs)	*In-silico* mutation starting from the X-ray structure of wild-type Rad6 (pdb entry 1AYZ[2])
Rad6-S120A	*S.cerevisiae*	Cys87<br><br>CES/D site: Ala119	4 replicates x 160 ns each (total duration 0.64 µs)	*In-silico* mutation starting from the X-ray structure of wild-type Rad6 (pdb entry 1AYZ[2])
hHR6B	Human	Cys87<br><br>CES/D site: Ser119	4 replicates x 160 ns each (total duration 0.64 µs)	NMR structure of wild-type hHR6B (pdb entry 1JAS[1])
Ubc1	*S.cerevisiae*	Cys88<br><br>CES/D site:	4 replicates x 160 ns each (total duration	X-ray structure of wild-type Ubc1 (pdb entry 1FZY[3])

		Ser120	0.64 μs)	
Ube2I	Human	Cys93<br><br>CES/D site: Asp127	4 replicates x 160 ns each (total duration 0.64 μs)	X-ray structure of wild-type Ube2I (pdb entry 1A3S[4])
Ube2I-SUB	Human	Cys93<br><br>CES/D site: Asp127	2 replicates x 80 ns each (total duration 0.16 μs)	X-ray structure of wild-type Ube2I in complex with RanGAP (PDB entry 2GRN[5])
$Ube2I_{D127S}$-SUB	Human	Cys93<br><br>CES/D site: Ser127	2 replicates x 80 ns each (total duration 0.16 μs)	X-ray structure of D127S Ube2I variant in complex with RanGAP (PDB entry 2GRR[5])
$Ube2I_{pS127}$-SUB	Human	Cys93<br><br>CES/D site: pSer127	2 replicates x 80 ns each (total duration 0.16 μs)	*In-silico* mutation starting from the X-ray structure of D127S Ube2I variant in complex with RanGAP (PDB entry 2GRR[5])
$Ube2I_{D127A}$-SUB	Human	Cys93<br><br>CES/D site: Ala127	2 replicates x 80 ns each (total duration 0.16 μs)	X-ray structure of D127A Ube2I variant in complex with RanGAP (PDB entry 2GRQ[5])
$Ube2I_{D127T}$-SUB	Human	Cys93<br><br>CES/D site: Thr127	2 replicates x 80 ns each (total duration 0.16 μs)	*In-silico* mutation starting from the X-ray structure of wild-type Ube2I variant in complex with RanGAP (PDB entry 2GRN[5])
$Ube2I_{D127E}$-SUB	Human	Cys93<br><br>CES/D site: Glu127	2 replicates x 80 ns each (total duration 0.16 μs)	*In-silico* mutation starting from the X-ray structure of wild-type Ube2I variant in complex with RanGAP (PDB entry 2GRN[5])

References

1. Miura, T., Klaus, W., Ross. A., Guentert, P. & Senn, H. The NMR structure of the class I human ubiquitin-conjugating enzyme 2b. *J. Biomol. NMR.* **22**, 89-92 (2002).
2. Worthylake, D.K., Prakash, S., Prakash, L. & Hill, C.P. Crystal structure of the Saccharomyces cerevisiae ubiquitin-conjugating enzyme Rad6 at 2.6 A resolution. *J. Biol.Chem.* **273**: 6271-6276 (1998).
3. Hamilton, K.S. et al. Crystal structure of Saccharomyces cerevisiae ubiquitin conjugating enzyme 1. **9**: 897-904 (2001).
4. Giraud, M.F., Desterro, J.M. & Naismith, J.H. Structure of ubiquitin-conjugating enzyme 9 displays significant differences with other ubiquitin-conjugating enzymes, which may reflect its specificity for Sumo rather than ubiquitin. *Acta Crystallogr. Sect D.* **54**: 891-898 (1998).
5. Yunus, A.A. & Lima, C. D. Lysine activation and functional analysis of E2-mediated conjugation in the SUMO pathway. *Nat. Struct. Mol. Biol.* **13,** 491–9 (2006).

Table S3. Hydrogen bond and salt bridges observed in the Ube2I-RanGAP1 simulations between the residue at position 127 (CES/D site) and the substrate acceptor lysine (Lys524). The Ube2I$_{D127A}$-RanGAP1 is not reported in this table since due to the hydrophobic nature of its side chain, an alanine residue at the CES/D site cannot be involved in electrostatic or hydrogen-bond interactions. Hydrogen bonds have been calculated using g_hbond tool of Gromacs, whereas salt bridges have been calculated using PyInteraph (http://linux.btbs.unimib.it/pyinteraph/). Hydrogen bonds are thus defined using a angle cutoff between donor-hydrogen-acceptor atoms of 30° and a distance cutoff of 0.35 nm between donor-acceptor atoms. Salt bridges have been defined with a distance cutoff of 0.45 nm between the charged groups of the two residues forming the salt bridge, using PyInteraph default definition.

**E2 variant**	***Pair of interacting residues***	**Salt-bridge population**	**Hydrogen Bond population**
Ube2I$_{pS127}$-RanGAP1	pS127-K524	100%	100%
Ube2I-RanGAP1	D127-K524	45%	23%
Ube2I$_{D127E}$-RanGAP1	E127-K524	74%	30%
Ube2I$_{D127S}$-RanGAP1	S127-K524	/	7%
Ube2I$_{D127T}$-RanGAP1	T127-K524	/	0.4 %

Figure S1. Multiple sequence alignment of E2 superfamily members used for the phylogenetic analysis. Identical residues (red-filled-boxes) and similar residues (red boxes) are indicated.

```
                                    α1                    η1     β1                  β2
Sc_Ubc1_1                                                                                TT
Sc_Ubc1_1         1  ....MS....RAKR.IMKEIQAVK.....DDPA.....AHITLEFV.SES....DIHHLKGTFLGP.PGT
Sp_Ubc1_1         1  ....MSD..NRSRR.IAKELADVQ.....QDKQ.....AGIQVWTI.ND.....DISHLKGMFRGP.EGT
Hs_UBE2K_1        1  ....MAN..IAVQR.IKREFKEVLKS...EETSK....NQIKVDLV.DE.....NFTELRGEIAGP.PDT
Os_Ubc_1          1  ....MVD..VS..R.VQKELTECNR....DREVS....GVSIALH.DGS.....TISHLTGTIAGP.QGT
Ce_Ubc20_1        1  ....MSN..IAFNR.LQRECKEVVT....NSEISE...TGIMIEIL.NE.....NLTEIKGHIRGP.PDT
Ce_Ubc1_2         1  ....MTT..PSRRR.LMRDFKKLQE....DPP......AGVSGAPT.ED.....NILTWEAIIFGP.QET
Hs_UBE2A_2        1  ....MST..PARRR.LMRDFKRLQE....DPP......AGVSGAPS.EN.....NIMVWNAVIFGP.EGT
Dm_UbcD6_2        1  ....MST..PARRR.LMRDFKRLQE....DPP......TGVSGAPT.DN.....NIMIWNAVIFGP.HDT
At_Ubc1_2         1  ....MST..PARKR.LMRDFKRLQQ....DPP......AGISGAPQ.DN.....NIMLWNAVIFGP.DDT
At_Ubc3_2         1  ....MTT..PAKKR.LMWDFKRLQK....DPP......VGISGAPQ.DN.....NIMHWNALIFGP.EDT
Sc_Ubc2_2         1  ....MST..PARRR.LMRDFKRMKE....DAP......PGVSASPL.PD.....NVMVWNAMIIGP.ADT
Nh_Rad6_2         1  ....MST..AARRR.LMRDFKRMQT....DPP......AGVSASPV.PD.....NVMTWNAVIIGP.ADT
Sp_Ubc2_2         1  ....MST..TARRR.LMRDFKRMQQ....DPP......AGVSASPV.SD.....NVMLWNAVIIGP.ADT
Sc_Ubc3_3         1  ....MSSRKSTASSLLLRQY.RELT....DPKKAI...PSFHIELE.DDS....NIFTWNIGVMVLNEDS
Hs_Ube2R1_3       1  ..MARPLVPSSQKA.LLLELKGLQE....EPV......EGFRVTLV.DEG....DLYNWEVAIFGP.PNT
Ce_Ubc3_3         1  MDSKASTSSGALRA.LTMELKNLQS....QPV......EGFTID.VNED.....NLFVWTVGIYGP.PKT
Sc_Ubc7_3         1  ....MSKTA..QKR.LLKELQQLIK....DSP......PGIVAGPKSEN.....NIFIWDCLIQGP.PDT
Mm_Ube2G2_3       1  ....MAGT...AKR.LMAEYKQLTL....NPP......EGIVAGPMNEE.....NFFEWEALIMGP.EDT
Ce_Ubc7_3         1  ....ME...QSSLL.LKKQLADMRR....VPV......DGFSAGLV.DD....NDIYKWEVLVIGP.PDT
Hs_Ube2G1_3       1  ....MTEL.QSALL.LRRQLAELNK....NPV......EGFSAGLI.DD....NDLYRWEVLIIGP.PDT
Ta_Ubc7_3         1  ....MATAPARRAS.SSRSSSEISRT...TPS......MGFQLGFV.DDS....NVFEWQVTIIGP.PET
Sp_Ubc15_3        1  ....MPSS.ASEQL.LRKQLKEIQK....NPP......QGFSVGLV.DDK....SIFEWEVMIIGP.EDT
Asfv_ubcB_3       1  .........MVSSF.LLAEYKNLIV....NPS......EHFKIS.VNED.....NLTEWDVILKGP.PDT
Ec_Ubc_3          1  ....MANKGGFSKG.LLLKDYKRMQE....NPN.....EYYSVGLV.DG.....DIYTWEVMIFGP.RKT
Hs_UBE2E1_4       1  .......STSA.KR.IQKELADITL....DPPPN.....CSAGPKGDN......IYEWRSTILGP.PGS
Sc_Ubc4_4         1  ........MSSSKR.IAKELSDLER....DPPTS.....CSAGPVGDD......LYHWQASIMGP.ADS
Ce_Ubc2_4         1  ........MA.LKR.IQKELQDLGR....DPPAQ.....CSAGPVGDD......LFHWQATIMGP.PES
Xl_Ubc4_4         1  ........MA.LKR.IHKELNDLAR....DPPAQ.....CSAGPVGDD......MFHWQATIMGP.NDS
Hs_Ubc2D2_4       1  ........MA.LKR.IHKELNDLAR....DPPAQ.....CSAGPVGDD......MFHWQATIMGP.NDS
Mm_MGC58426_4     1  ........MA.LKR.IQKELVAISQ....DPPAH.....CSAGPVAEN......MFHWQATIMGP.EDS
Dm_CG5440_4       1  .....TCSNSAVKR.IQKELDEITR....DPPQY.....CSAGPKEDN......LYEWTSTIIGP.ADS
Dm_CG10862_4      1  .......PLT.ITR.LRREISEFST....DQTEG.....CKAEMVGDN......LFHWVATIPGP.SET
Dm_CG2574_4       1  ........TGCVVR.IKSELQDIRK....NPPPN.....CTADLHHGD......LLHWTAGVNGP.VGS
Hs_UBE2J2_5       1  .........TATQR.LKQDYLRIKK....DPVPY.....ICAEPL.PS.....NILEWHYVVRGP.EMT
Sc_Ubc6_5         1  ..........AHKR.LTKEYKLMVE....NPPPY.....ILARPN.ED.....NILEWHYIITGP.ADT
Ce_Ubc15_5        1  .........SAVRR.LQKDYAKLMQ....DPVDG.....IKALPN.ED.....NILEWHYCLRGS.PDT
Pm_Sb55_5         1  .........ACVKR.LQKEFRALCK....EPVPN.....VVARPS.PS.....DILEWHYVLEGS.EGT
At_Ubc32_5        1  .......KNPAVKR.ILQEVKEMQA....NPSDD.....FMSLPL.EE.....NIFEWQFAIRGP.GDT
Hs_UBE2J1_5       1  .........PAVKR.LMKEAAELK.....DPTDH.....YHAQPL.ED.....NLFEWHFTVRGP.PDS
Hs_UBE2H_6        1  ...MSSPS.PGKRR.MDTDVVKLIE....SKHEV......TILGG.........LNEFVVKFYGP.QGT
Ce_Ubc8_6         1  ...MTSATAIGKRR.IDCDVVKLIS....HNHEV......QIVNG.........CSEFIVRFHGP.KDT
At_Ubc6_6         1  ...MASP...SKRR.EMDMMKLMM.....SDYKV......DTVN..DD......LQMFYVTFHGP.TDS
Sc_Ubc8_6         1  ...MSS....SKRR.IETDVMKLLM....SDHQV......DLIN..DS......MQEFHVKFLGP.KDT
Sp_Ubc8_6         1  ...MSSP....RRR.IETDVMKLLM....SDYEV......TLVN..DN......MQEFYVRFHGP.SET
Dm_CG14739_6      1  ..LVNTTLPMAGRR.LDRDVNRLLA....SGYR.......TTVD..DD......MTNLNVCLEGP.LGS
Sc_Ubc9_7         1  .....MSS.LCLQR.LQEERKKWRK....DHP......FGFYAKPV.KKADGSMDLQKWEAGIPGK.EGT
Mm_Ube2I_7        1  .....MSG.IALSR.LAQERKAWRK....DHP......FGFVAVPT.KNPDGTMNLMNWECAIPGK.KGT
Hs_Ube2I_7        1  .....MSG.IALSR.LAQERKAWRK....DHP......FGFVAVPT.KNPDGTMNLMNWECAIPGK.KGT
Sp_Ubc9_7         1  .....MSS.LCKTR.LQEERKQWRR....DHP......FGFYAKPC.KSSDGGLDLMNWKVGIPGK.PKT
At_AHUS5_7        1  .....MASGIARGR.LAEERKSWRK....NHP......HGFVAKPE.TGQDGTVNLMVWHCTIPGK.AGT
Hs_Ube2F_8        1  ......RVSVRDKL.LVKEVAELEA....NLPCT.....CKVHF.PDP.....NKLHCFQLTVTP.DEG
Ce_Ubc12_8        1  ......RIAVRDKL.LAQELQQLET....ALRDQK....QKLWHLEVPST....SCLHELELTVTP.QEG
Hs_Ube2M_8        1  ......KASAAQLR.IQKDINELNL....PKTCD.....ISFSD..PD.....DLLN.FKLVICP.DEG
Sp_Ubc12_8        1  .......ISPAQIR.IQKDVTDLEI....PSTMS.....TSWPD..PI.....KLNV.LHLEIRP.DEG
At_RCE2_8         1  ......KQSAGELR.LHKDISELNL....PKSCK.....ISFPNG.KN.....DLMN.FEVTIKP.DEG
Nc_Ubc_8          1  ......KVTAAQLR.VQKDLSELSL....GSTMK.....TEFPD..PD.....DILN.FILYIEP.DEG
Sc_Ubc12_8        1  ......NLSAARIR.LKRDLDSLDL....PPTVTL....NVIT..SPDSADRSQSPK.LEVIVRP.DEG
Hs_UBE2T_9        1  .......MQ.RASR.LKRELHMLAT....EPPP.....GITCWQ.DKD.....QMDDLRAQILGG.ANT
Sc_Ubc13_9        1  .......MASLPKR.IIKETEKLVS....DPVP.....GITAEPH.DD.....NLRYFQVTIEGP.EQS
Hs_UBE2N_9        1  .......MAGLPRR.IIKETQRLLA....EPVP.....GIKAEPD.ES.....NARYFHVVIAGP.QDS
Lm_Ubc_9          1  .......M..LTTR.IIKETEKLQK....ECPP.....GITATPT.KE.....NPRYFMVTIQGP.PQS
Hs_UBE2S_11       1  .....ENLPPHIIRLVYKEVTTLTA....DPP.....DGIKVFPN.EE.....DLTDLQVTIEGP.EGT
At_UBC22_11       1  ....ENLPPNVIKQ.LAKELK.SLD....ESPP....DGIKVVVN.DE.....DFSQICADIEGP.VGT
Sc_Ubc10_11       1  ......TSDTCMSR.IVKEYKVILKTLASDDPIANPYRGIIESLNPIDET....DLSKWEAIISGP.SDT
Sp_UBC16_11       1  .......MNSSIKR.LHKEYASFQA....GDI......SGLLLLKPVTDV....DMFHWKAVIEGP.TET
Sc_Ubc11_12       1  ..........CVTKR.LQNELLQLLS....STT.....ESISAFP.VDD....NDLTYWVGYITGP.KDT
Ss_E2C_12         1  ..........SVSKR.LQQELRTLLM....SGD.....PGITAFP.DGD.....NLFKWVATLDGP.KDT
Hs_UBE2C_12       1  ..........PVGKR.LQQELMTLMM....SGD.....KGISAFP.ESD.....NLFKWVGTIHGA.AGT
Dm_VIHAR_12       1  ..........AVSKR.LHKELMNLMM....ANE.....RGISAFP.DGE.....NIFKWVGTIAGP.RNT
At_Ubc19_12       1  ..........SVLKR.LQSELMGLMM....GAD.....PGISAFP.EED.....NIFCWKGTITGS.KDT
Hs_UBE2W_13       1  ......MA.SMQKR.LQKELLALQN....DPPP....GMTLNEKSVQN.....SITQWIVDMEGA.PGT
Ce_Ubc16_13       1  ......MSDAATRR.LMKELAQLKS....EAPE....GLLVDNTSTSN.....DLKQWKIGVVGA.EGT
At_UBC15_13       1  ...RKALSKIACNR.LQKELSEWQL....NPPT....GFRHKVTD........NLQKWTIDVTGA.PGT
Hs_BIRC6_14       1  ......SAARA.RR.LAQEAVTLST....SLP..LSSSSSVFVRC.DEE....RLDIMKVLITGP.ADT
Ce_Ubc17_14       1  ......HLRDRTKR.IAKELASIAN....ALP..LNASNSIYVCY.DEG....RVDIIKVLISGP.DDT
At_Ubc23_14       1  ......KERQWFKK.VDQDWKILQN....NLP.....DGIFVRA.YED.....RMDLLRAVIVGA.FGT
Hs_UBE2O_14       1  ......EAKKFFST.VRKEMALLAT....SLP.....EGIMVKT.FED.....RMDLFSALIKGP.TRT
Dm_CG10254_14     1  .....ANKAQYQRA.VQREYLMLKS....SLP.....NGVVVRA.YED.....RMDLMSVMMVGP.KRT
Hs_UBE2Z_14       1  .....RTAPQCLLR.IKRDIMSIYK....EPP.....PGMFVVP.DTV.....DMTKIHALITGP.FDT
Hs_UBE2L6_15      1  ........MMASMR.VVKELEDLQK....PPPY.L......RNLSSDDA.....NVLVWHALLL.P.DQP
Hs_UBE2L3_15      1  ........MAASRR.LMKELEEIRK....CGMKNF......RNIQVDEA.....NLLTWQGLIV.P.DNP
Ce_Ubc18_15       1  ........MSATRR.LQKELGDLKN....CGVKAY......ENVECEET.....NLLKWTVLLI.P.DKE
Dm_CG17030_15     1  ....EEPLMDGPKR.MNRELALMLE....DKQNLQF.....RNLLVEPN.....NIYKWTGLLM.P.VAP
Ce_Ubc24_15       1  ...AAHGRIVQLSR.IRKEIADLAK....NKRR.....FIKDFRKIEK......CKDIFQFKIIGD..GV
Dm_UbcD84_15      1  ........MAATRR.LTRELSDLVE....AKMSTL......RNIESSDE.....SLLMWTGLLV.P.EKA
```

```
                        β3               β4                    η2
Sc_Ubc1_1                                              TT  TTT
Sc_Ubc1_1       46  PYEGGKFVVDIEVPMEYPFKPPKMQFDT.....KVYHPNISSVTGAICLDILKN.............AWS
Sp_Ubc1_1       47  PYEGGYFVVDIEIPIDYPFRPPKMNFDT.....KIYHPNVSSQTGAICLDILKD.............QWS
Hs_UBE2K_1      50  PYEGGRYQLEIKIPETYPFNPPKVRFIT.....KIWHPNISSVTGAICLDILKD.............QWA
Os_Ubc_1        47  PYEGGTFVIDIRLPGGYPFEPPKMQFIT.....KVWHPNISSQNGAICLDILKD.............QWS
Ce_Ubc20_1      50  PYAGGMFDLDIKIPDQYPFSPPNVKFST.....KIWHPNVSSQTGVICLDILKD.............QWA
Ce_Ubc1_2       47  PFEDGTFKLSLEFTEEYPNKPPTVKFIS.....KMFHPNVY.ADGSICLDILQN.............RWS
Hs_UBE2A_2      47  PFEDGTFKLTIEFTEEYPNKPPTVRFVS.....KMFHPNVY.ADGSICLDILQN.............RWS
Dm_UbcD6_2      47  PFEDGTFKLTIEFTEEYPNKPPTVRFVS.....KVFHPNVY.ADGGICLDILQN.............RWS
At_Ubc1_2       47  PWDGGTFKLSLQFSEDYPNKPPTVRFVS.....RMFHPNIY.ADGSICLDILQN.............QWS
At_Ubc3_2       47  PWDGGTFKLTLHFTEDYPNKPPIVRFVS.....RMFHPNIY.ADGSICLDILQN.............QWS
Sc_Ubc2_2       47  PYEDGTFRLLLEFDEEYPNKPPHVKFLS.....EMFHPNVY.ANGEICLDILQN.............RWT
Nh_Rad6_2       47  PFEDGTFRLVMQFEEQYPNKPPQVKFIS.....EMFHPNVY.ATGELCLDILQN.............RWS
Sp_Ubc2_2       47  PFEDGTFKLVLSFDEQYPNKPPLVKFVS.....TMFHPNVY.ANGELCLDILQN.............RWS
Sc_Ubc3_3       54  IYHGGFFKAQMRFPEDFPFSPPQFRFTP.....AIYHPNVY.RDGRLCISILHQSG.DPMTDEPDAETWS
Hs_Ube2R1_3     52  YYEGGYFKARLKFPIDYPYSPPAFRFLT.....KMWHPNIY.ETGDVCISILHPPVDDPQSGELPSERWN
Ce_Ubc3_3       53  LYQGGYFKASIRFPSNYPYSPPSMKFTT.....KVWHPNVY.ENGDLCISILHSPIDDPQSGELACERWN
Sc_Ubc7_3       48  PYADGVFNAKLEFPKDYPLSPPKLTFTP.....SILHPNIY.PNGEVCISILHSPGDDPNMYELAEERWS
Mm_Ube2G2_3     47  CFEFGVFPAILSFPLDYPLSPPKMRFTC.....EMFHPNIY.PDGRVCISILHAPGDDPMGYESSAERWS
Ce_Ubc7_3       47  LYEGGFFKAILDFPRDYPQKPPKMKFIS.....EIWHPNID.KEGNVCISILHDPGDDKWGYERPEERWL
Hs_Ube2G1_3     49  LYEGGVFKAHLTFPKDYPLRPPKMKFIT.....EIWHPNVD.KNGDVCISILHEPGEDKYGYEKPEERWL
Ta_Ubc7_3       51  LYDGGYFNAIMSFPQNYPNSPPTVRFTS.....EMWHPNVY.PDGRVCISI.HPPGDDPNGYELASERWT
Sp_Ubc15_3      49  LYEGGFFHATLSFPQDYPLMPPKMKFTT.....EIWHPNVH.PNGEVCISILHPPGDDKYGYEDAGERWL
Asfv_ubcB_3     44  LYEGGLFKAKIVFPPKYPYEPPRLTFTS.....EMWHPNIY.SDGKLCISILH..G.DNA..EEQGMTWS
Ec_Ubc_3        50  PYENGIFKGRMLFPTDYPDSPPKFRFCS.....KMWHPNID.ENGNVCISILHNPGEDEYGYESLGDRWL
Hs_UBE2E1_4     45  VYEGGVFFLDITFTPEYPFKPPKVTFRT.....RIYHCNINS.QGVICLDILKD.............NWS
Sc_Ubc4_4       45  PYAGGVFFLSIHFPTDYPFKPPKISFTT.....KIYHPNINA.NGNICLDILKD.............QWS
Ce_Ubc2_4       44  PYQGGVFFLTIHFPTDYPFKPPKVAFTT.....RIYHPNINS.NGSICLDILRS.............QWS
Xl_Ubc4_4       44  PYQGGVFFLTIHFPTDYPFKPPKVAFTT.....RIYHPNINS.NGSICLDILRS.............QWS
Hs_Ubc2D2_4     44  PYQGGVFFLTIHFPTDYPFKPPKVAFTT.....RIYHPNINS.NGSICLDILRS.............QWS
Mm_MGC58426_4   44  PYQGGVFFLSVHFPNNYPFKPPKVTFIT.....RVYHPNISK.NGSICLDILNS.............MWS
Dm_CG5440_4     48  VYENGIFKLDIFFPVEYPFAPPVVIFRT.....PIYHCNIHR.LGFICLDILKE.............KWS
Dm_CG10862_4    45  VYEGGRFRVEIVFPRNYPFYPPYLAFLT.....KTYHCNIAL.SGRICLDILGS.............KWS
Dm_CG2574_4     45  VYEGGHFRLDIRFPASYPFRAPRIRFTT.....RIYHCNVDS.RGAICLDVLGE.............RWS
Hs_UBE2J2_5     44  PYEGGYYHGKLIFPREFPFKPPSIYMIT........PNGRFKCNTRLCLSITDFHP..........DTWN
Sc_Ubc6_5       43  PYKGGQYHGTLTFPSDYPYKPPAIRMIT........PNGRFKPNTRLCLSMSDYHP..........DTWN
Ce_Ubc15_5      44  PFYGGYYWGKVIFKENFPWSPPAITMIT........PNGRFQTNTRLCLSISDYHP..........ESWN
Pm_Sb55_5       44  PFAGGVYHGKVKFPPEYPYKPPGISMIT........PNGRFATHKRICMSMSDYHP..........ETWN
At_Ubc32_5      46  EFEGGIYHGRIQLPADYPFKPPSFMLLT........PNGRFETNTKICLSISNYHP..........EHWQ
Hs_UBE2J1_5     43  DFDGGVYHGRIVLPPEYPMKPPSIILLT........ANGRFEVGKKICLSISGHHP..........ETWQ
Hs_UBE2H_6      45  PYEGGVWKVRVDLPDKYPFKSPSIGFMN.....KIFHPNIDEASGTVCLDVINQ.............TWT
Ce_Ubc8_6       46  AYENGVWRIRVDMPDKYPFKSPSIGFLN.....KIFHPNIDEASGTVCLDVINQ.............AWT
At_Ubc6_6       43  LYQGGVWKIKVELPEAYPYKSPSVGFVN.....KIYHPNVDESSGAVCLDVINQ.............TWS
Sc_Ubc8_6       43  PYENGVWRLHVELPDNYPYKSPSIGFVN.....KIFHPNIDIASGSICLDVINS.............TWS
Sp_Ubc8_6       43  PYSGGIWKVHVELPSEYPWKSPSIGFVN.....RIFHPNIDELSGSVCLDVINQ.............TWS
Dm_CG14739_6    47  AYEGGIWTVNVTMPQDYPLTAPRVRFVT.....KILHPNIEFITGLVCMNVLKQ.............AWS
Sc_Ubc9_7       52  NWAGGVYPITVEYPNEYPSKPPKVKFPA.....GFYHPNVY.PSGTICLSILNED...........QDWR
Mm_Ube2I_7      52  PWEGGLFKLRMLFKDDYPSSPPKCKFEP.....PLFHPNVY.PSGTVCLSILEED...........KDWR
Hs_Ube2I_7      52  PWEGGLFKLRMLFKDDYPSSPPKCKFEP.....PLFHPNVY.PSGTVCLSILEED...........KDWR
Sp_Ubc9_7       52  SWEGGLYKLTMAFPEEYPTRPPKCRFTP.....PLFHPNVY.PSGTVCLSILNEE...........EGWK
At_AHUS5_7      53  DWEGGFFPLTMHFSEDYPSKPPKCKFPQ.....GFFHPNVY.PSGTVCLSILNED...........YGWR
Hs_Ube2F_8      47  YYQGGKFQFETEVPDAYNMVPPKVKCLT.....KIWHPNITE.TGEICLSLLREHSIDG.......TGWA
Ce_Ubc12_8      51  IYRGGKFRFKITVPPEYNNVPPVVKCLT.....KVWHPNINE.DGSICLSILRQNSLDQ.......YGWR
Hs_Ube2M_8      45  FYKSGKFVFSFKVGQGYPHDPPKVKCET.....MVYHPNIDL.EGNVCLNILRE.............DWK
Sp_Ubc12_8      44  YYKGGKFKFRIQIDDNYPHDPPKVKCLN.....KIYHPNIDI.EGNVCLNILRQ.............DWN
At_RCE2_8       46  YYLSGNFVFSFQVSNMYPHEAPKVKCKT.....KVYHPNIDL.EGNVCLNILRE.............DWK
Nc_Ubc_8        45  MYKGGKFSFTFNITPNFPHEPPKVQCRE.....KIYHPNIDL.EGKVCLNILRE.............DWK
Sc_Ubc12_8      51  YYNYGSINFNLDFNEVYPIEPPKVVCLK.....KIFHPNIDL.KGNVCLNILRE.............DWS
Hs_UBE2T_9      45  PYEKGVFKLEVIIPERYPFEPPQIRFLT.....PIYHPNIDS.AGRICLDVLKLPPKG.........AWR
Sc_Ubc13_9      46  PYEDGIFELELYLPDDYPMEAPKVRFLT.....KIYHPNIDR.LGRICLDVLKT.............NWS
Hs_UBE2N_9      46  PFEGGTFKLELFLPEEYPMAAPKVRFMT.....KIYHPNVDK.LGRICLDILKD.............KWS
Lm_Ubc_9        44  CYEGGLFRLELFLPEEYPMKPPKVRFLT.....RIYHPNVDK.VGRICLDIIKD.............KWS
Hs_UBE2S_11     49  PYAGGLFRMKLLLGKDFPASPPKGYFLT.....KIFHLNVGA.NGEICVNVLK............RDWT
At_UBC22_11     49  PYENGLFRMKLALSHDFPHSPPKGYFMT.....KIFHPNVAS.NGEICVNTLK............KDWN
Sc_Ubc10_11     59  PYENHQFRILIEVPSSYPMNPPKISFMQN....NILHCNVKSATGEICLNILKPE.............EWT
Sp_UBC16_11     48  PYEGGQWVLDIHVHEGYPISPPSVYFQT.....KIVHPNISWTNGEVCMDILKT.............HWS
Sc_Ubc11_12     45  PYSGLKFKVSLKFPQNYPFHPPMIKFLS.....PMWHPNVD.KSGNICLDILK............EKWS
Ss_E2C_12       44  VYESLKYKLTLEFPSDYPYKPPVVKFTT.....PCWHPNVD.QSGNICLDILK............ENWT
Hs_UBE2C_12     44  VYEDLRYKLSLEFPSGYPYNAPTVKFLT.....PCYHPNVD.TQGNICLDILK............EKWS
Dm_VIHAR_12     44  VYSGQTYRLSLDFPNSYPYAAPVVKFLT.....SCFHPNVD.LQGAICLDILK............DKWS
At_Ubc19_12     44  VFEGTEYRLSLTFSNDYPFKSPKVKFET.....CCFHPNVD.LYGNICLDILQ............DKWS
Hs_UBE2W_13     48  LYEGEKFQLLFKFSSRYPFDSPQVMFTGEN...IPVHPHVYS.NGHICLSILTE.............DWS
Ce_Ubc16_13     49  LYAGEVFMLQFTFGPQYPFNSPEVMFVGET...IPAHPHIYS.NGHICLSILSD.............DWT
At_UBC15_13     49  LYANETYQLQVEFPEHYPMEAPQVVFVSP....APSHPHIYS.NGHICLDILYD.............SWS
Hs_BIRC6_14     50  PYANGCFEFDVYFPQDYPSSPPLVNLETTGGHSVRFNPNLYN.DGKVCLSILNTWHGRPE.....EKWN
Ce_Ubc17_14     51  PYANGLFEFDIFFPTGYPFSPPKCAFLTTGSGNVRFNPNLYN.DGKICLSILGTWEGRPE.....EKWN
At_Ubc23_14     47  PYQDGLFFFDFHLPSDYPSVPPSAYYHS...GGWRLNPNLYE.EGKVCLSLLNTWTGRGN.....EVWD
Hs_UBE2O_14     47  PYEDGLYLFDIQLPNIYPAVPPHFCYLSQ..CSGRLNPNLYD.NGKVCVSLLGTWIGKGT.....ERWT
Dm_CG10254_14   48  PYQNALFFFDFQFGRDYPKSPPVCHYISY..CTDRLNPNLYE.GGRVCVSLLGTWMGRDN.....EVWS
Hs_UBE2Z_14     48  PYEGGFFLFVFRCPPDYPIHPPRVKLMTTGNNTVRFNPNFYR.NGKVCLSILGTWTG........PAWS
Hs_UBE2L6_15    44  PYHLKAFNLRISFPPEYPFKPPMIKFTT.....KIYHPNVDE.NGQICLPIISSE............NWK
Hs_UBE2L3_15    45  PYDKGAFRIEINFPAEYPFKPPKITFKT.....KIYHPNIDE.KGQVCLPVISAE............NWK
Ce_Ubc18_15     45  PYNKGAFKVGITFPVDYPFKPPKVAFET.....KIYHPNVDE.EGKFCLPIVTAE............NWK
Dm_CG17030_15   50  PYDKGAYKMEIDFPLDYPFKPPRIHINT.....RMYHLNVNE.RGQVCVPILEVE............HWI
Ce_Ubc24_15     50  LYKNMIFTLTLDVNVEYPFKPPYLKFCH.....NVYHPNVDPVTCELCSPMLLQE............NWK
Dm_UbcD84_15    45  PYNKGAFRIEINFPPQYPFMPPKILFKT.....KIYHPNVDE.KGEVCLPIISTD............NWK
```

```
                                          α2                                α3                         α4
Sc_Ubc1_1                TT   . llllllllll.lll       TT .    lllllllll                  llllllllllllllll

Sc_Ubc1_1          98    PVIT.LKSALISLQA.LLQSP..EPNDPQDAEVAQHYLR...D.....RESFNKTAALWTRLYAS..
Sp_Ubc1_1          99    PVYT.MKSALISLQS.LLCTP..EPSNPQDAQVAQVYLQ...N.....YQQFVRTAREWTSSYAA..
Hs_UBE2K_1        102    AAMT.LRTVLLSLQA.LLAAA..EPDDPQDAVVANQYKQ...N.....PEMFKQTARLWAHVYAG..
Os_Ubc_1           99    PALT.LKTALLSLQA.LLSAP..APDDPQDAVVAQQYLR...D.....YSTFSATARYWTEAFAK..
Ce_Ubc20_1        102    ASLT.LRTVLLSIQA.LMCTP..EPKDPQDAVVAKQYME...K.....PAVFKATAEYWTTKFAK..
Ce_Ubc1_2          98    PTYD.VAAILTSIQS.LLDEP..NPNSPANSLAAQLYQE...N.....RREYEKRVQQIVEQSWLNF
Hs_UBE2A_2         98    PTYD.VSSILTSIQS.LLDEP..NPNSPANSQAAQLYQE...N.....KREYEKRVSAIVEQSWRDC
Dm_UbcD6_2         98    PTYD.VSAILTSIQS.LLSDP..NPNSPANSTAAQLYKE...N.....RREYEKRVKACVEQSFID.
At_Ubc1_2          98    PIYD.VAAILTSIQS.LLCDP..NPNSPANSEAARMYSE...S.....KREYNRRVRDVVEQSWTAD
At_Ubc3_2          98    PIYD.VAAVLTSIQS.LLCDP..NPDSPANAEAARLFSE...N.....KREYNRKVIEIVEQSYV..
Sc_Ubc2_2          98    PTYD.VASILTSIQS.LFNDP..NPASPANVEAATLFKD...H.....KSQYVKRVKETVEKSWEDD
Nh_Rad6_2          98    PTYD.VAAVLTSIQS.LLNDP..NTGSPANVEASNLYKD...N.....RKEYTKRVRETVEKSWED.
Sp_Ubc2_2          98    PTYD.VAAILTSIQS.LLNDP..NNASPANAEAAQLHRE...N.....KKEYVRRVRKTVEDSWES.
Sc_Ubc3_3         117    PVQT.VESVLISIVS.LLEDP..NINSPANVDAAVDYRK...N.....PEQYKQRVKMEVERSKQD.
Hs_Ube2R1_3       116    PTQN.VRTILLSVIS.LLNEP..NTFSPANVDASVMYRK..WKESKGKDREYTDIIRKQVLGTKVD.
Ce_Ubc3_3         117    PTQS.VRTILLSVIS.LLNEP..NTSSPANVDASVMYRK..WKEDQ..DPEYAKIVTKQVEESKKV.
Sc_Ubc7_3         112    PVQS.VEKILLSVMS.MLSEP..NIESGANIDACILWRD...N.....RPEFERQVKLSILKSLGF.
Mm_Ube2G2_3       111    PVQS.VEKILLSVVS.MLAEP..NDESGANVDASKMWRD...D.....REQFYKIAKQIVQKSLGL.
Ce_Ubc7_3         111    PVHT.VETILLSVIS.MLTDP..NFESPANVDAAKMQRE...N.....YAEFKKKVAQCVRRSQEE.
Hs_Ube2G1_3       113    PIHT.VETIMISVIS.MLADP..NGDSPANVDAAKEWRE...DR....NGEFKRKVARCVRKSQET.
Ta_Ubc7_3         114    PVHT.VESIVLSIIS.MLSSP..NDESPANIEAAKDWRE...K.....QDEFKKKVRRAVRKSQEM.
Sp_Ubc15_3        113    PVHS.PETILISVIS.MLSSP..NDESPANIDAAKEFRE...N.....PQEFKKRVRRLVRRSIEM.
Asfv_ubcB_3       103    PAQK.IDTVLLSVIS.LLNEP..NPDSPANVDAAKSYRKYLYKEDL...ESYPMEVKKTVKKSLDE.
Ec_Ubc_3          114    PVRT.PESVILSIIT.LLTSP..NCESPANVDAAQHLRE...N.....EREYRKKVMELAAMTLDL.
Hs_UBE2E1_4        96    PALT.ISKVLLSICS.LLTDC..NPADPLVGSIATQYMT...N.....RAEHDRMARQWTKRYAT..
Sc_Ubc4_4          96    PALT.LSKVLLSICS.LLTDA..NPDDPLVPEIAHIYKT...D.....RPKYEATAREWTKKYAV..
Ce_Ubc2_4          95    PALT.ISKVLLSICS.LLCDP..NPDDPLVPEIARIYKT...D.....RERYNQLAREWTQKYAM..
Xl_Ubc4_4          95    PALT.ISKVLLSICS.LLCDP..NPDDPLVPEIARIYKT...D.....REKYNRIAREWTQKYAM..
Hs_Ubc2D2_4        95    PALT.ISKVLLSICS.LLCDP..NPDDPLVPEIARIYKT...D.....REKYNRIAREWTQKYAM..
Mm_MGC58426_4      95    PALT.ISKLLLSICS.LLCDP..NPDDPLVPEIAKVYRK...D.....LREYNRLAREWTKRYAM..
Dm_CG5440_4        99    PALT.ISKILLSICS.LLTDC..NPKDPLMAKIGTEYLK...N.....RAEHDKKARLWTKR.....
Dm_CG10862_4       96    PALS.VSKVLISIMS.LLADP..NPHDPMEVSVADVFKG...N.....RALHDKNAREWTKKYAK..
Dm_CG2574_4        96    PVMN.VAKVLLSIYV.LMSEC..NPDDPLVMCIADQYKT...N.....RREHDKIARHWTKLFAM..
Hs_UBE2J2_5        96    PAWS.VSTILTGLLS..FMVE..KGPTLGSIET.................SDFTKRQLAVQS....
Sc_Ubc6_5          95    PGWS.VSTILNGLLS..FMTS..DEATTGSITT.................SDHQKKTLARNS....
Ce_Ubc15_5         96    PGWT.VSAILIGLHS..FMNE..NSPAAGSIAG.................TPQDQRMYAAAS....
Pm_Sb55_5          96    PMWS.VSSILTGLLS..FMMD..NSPTTGSVST.................TVADKQRLAKAS....
At_Ubc32_5         98    PSWS.VRTALVALIA..FMPT.SPNGALGSVDY.................PKDERRTLAIKS....
Hs_UBE2J1_5        95    PSWS.IRTALLAIIG..FMPT.KGEGAIGSLDY.................TPEERRALAKKSQDF.
Hs_UBE2H_6         97    ALYD.LTNIFESFLPQLLAYP..NPIDPLNGDAAAMYLH...R.....PEEYKQKIKEYIQKYAT..
Ce_Ubc8_6          98    ALYD.LTNIFDTFLPQLLTYP..NAADPLNGEAARLYIH...K.....PEEYRRTCREYVMRFASEH
At_Ubc6_6          95    PMFD.LINVFESFLPQLLLYP..NPSDPFNGEAASLLMR...D.....RAAYELKVKEYCEKYAKP.
Sc_Ubc8_6          95    PLYD.LINIVEWMIPGLLKEP..NGSDPLNNEAATLQLR...D.....KKLYEEKIKEYIDKYATKE
Sp_Ubc8_6          95    PMFD.MINIFEVFLPQLLRYP..NASDPLNGEAAALLLR...E.....PNTYYAKVRDYIARYA...
Dm_CG14739_6       99    SSYD.LVNIFETFLPQLLRYP..NPHDSLNHRAAAIMKH...S.....EQLFREHVILCMKTYAMPA
Sc_Ubc9_7         105    PAIT.LKQIVLGVQD.LLDSP..NPNSPAQEPAWRSFSR...N.....KAEYDKKVLLQAKQYSK..
Mm_Ube2I_7        105    PAIT.IKQILLGIQE.LLNEP..NIQDPAQAEAYTIYCQ...N.....RVEYEKRVRAQAKKFAPS.
Hs_Ube2I_7        105    PAIT.IKQILLGIQE.LLNEP..NIQDPAQAEAYTIYCQ...N.....RVEYEKRVRAQAKKFAPS.
Sp_Ubc9_7         105    PAIT.IKQILLGIQD.LLDDP..NIASPAQTEAYTMFKK...D.....KVEYEKRVRAQARENAP..
At_AHUS5_7        106    PAIT.VKQILVGIQD.LLDTP..NPADPAQTDGYHLFCQ...D.....PVEYKKRVKLQSKQYPALV
Hs_Ube2F_8        104    PTRT.LKDVVWGLNS.LFTDLL.NFDDPLNIEAAEHHLR...D.....KEDFRNKVDDYIKRYAR..
Ce_Ubc12_8        108    PTRN.LTDVVHGLVS.LFNDLM.DFNDALNIQAAQMWSQ...N.....RESFNHRVREYISRYC...
Hs_Ube2M_8         96    PVLT.INSIIYGLQY.LFLEP..NPEDPLNKEAAEVLQN...N.....RRLFEQNVQRSMRGGYIG.
Sp_Ubc12_8         95    PVLN.LNSILVGLQF.LFLSP..NAEDPLNKEAAADLHK...D.....PQGFASRVRTAMKGGLVN.
At_RCE2_8          97    PVLN.INTVIYGLFH.LFTEP..NYEDPLNHEAAAVLRD...N.....PKTFEYNVRRAMMGGQVG.
Nc_Ubc_8           96    PVLN.LNAVIVGLQF.LFLEP..NASDPLNKEAAEDLRS...N.....REGFKRNVRTAMSGGTVK.
Sc_Ubc12_8        102    PALD.LQSIITGLLF.LFLEP..NPNDPLNKDAAKLLCE...G.....EKEFAEAVRLTMSGGSIE.
Hs_UBE2T_9        100    PSLN.IATVLTSIQL.LMSEP..NPDDPLMADISSEFKY...N.....KPAFLKNARQWTEKHARQK
Sc_Ubc13_9         97    PALQ.IRTVLLSIQA.LLASP..NPNDPLANDVAEDWIK...N.....EQGAKAKAREWTKLYAK..
Hs_UBE2N_9         97    PALQ.IRTVLLSIQA.LLSAP..NPDDPLANDVAEQWKT...N.....EAQAIETARAWTRLYAM..
Lm_Ubc_9           95    PALL.INKVLLSIQI.LMSSP..NPDDPLANDVAEHWKE...D.....EASALQTAREWTRKYAK..
Hs_UBE2S_11       100    AELG.IRHVLLTIRC.LLIHP..NPESALNEEAGRLLLE...N.....SEEYAARARLLTEIHGG..
At_UBC22_11       100    PSLG.LRHVLSVVRC.LLIEP..FPESALNEQAGKMLLE...N.....YDEYARHARLYTGIHAK..
Sc_Ubc10_11       113    PVWD.LLHCVHAVWR.LLREP..VCDSPLDVDIGNIIRCG..D.....MSAYQGIVKYFLAERER..
Sp_UBC16_11       100    PAWS.LQSACLAIIS.LLSNY..DASSPLNVDAAKLLRTG..D.....KTAYNSLVRCTTYLYAK..
Sc_Ubc11_12        96    AVYN.VETILLSLQS.LLGEP..NNRSPLNAVAAELWDA...D.....MEEYRKKVLACYEEIDD..
Ss_E2C_12          95    ASYD.VRTILLSLQS.LLGEP..NNASPLNAQAADMWS....N.....QTEYKKVLHEKYKTAQS..
Hs_UBE2C_12        95    ALYD.VRTILLSIQS.LLGEP..NIDSPLNTHAAELWK....N.....PTAFKKYLQETYSKQVT..
Dm_VIHAR_12        95    ALYD.VRTILLSIQS.LLGEP..NNESPLNAQAAMMWN....D.....QKEYKKYLDAFYEKHKD..
At_Ubc19_12        95    SAYD.VRTILLSIQS.LLGEP..NISSPLNNQAAQLWS....N.....QEEYRKMVEKLYKPLNA..
Hs_UBE2W_13       101    PALS.VQSVCLSIIS.MLSSCK.EKRRPPDNSFYVRTCN.........KNPKKTKWWYHDDTC...
Ce_Ubc16_13       102    PALS.VQSVCLSILS.MLSSSK.EKKHPIDDAIYVRTCS.........KNPSKTRWWFHDDSV...
At_UBC15_13       101    PAMT.VNSVCISILS.MLSSSP.AKQRPADNDRYVKNCKN........GRSPKETRWWFHDDKV...
Hs_BIRC6_14       113    PQTSSFLQVLVSVQSLILVAEP.YFNEPGYERSRGTPSGT.QS.....SREYDGNIRQATVKWAM..
Ce_Ubc17_14       114    PYCS.LMQVLVSIQEVI.EKHLWLKREAILK.QAQAWID.NV.....SNDFGDDKMSKRKDVFA..
At_Ubc23_14       107    PKSSSILQVLVSLQGLVLNSKP.YFNEAGYDKQVGTAEGE.KN.....SLGYNENTFLLNCKTMM..
Hs_UBE2O_14       108    SKSS.LLQVLISIQGLILVNEP.YYNEAGFDSDRGLQEGY.EN.....SRCYNEMALIRVVQSMT..
Dm_CG10254_14     109    PSST.MLQVLVSIQGLILVDEP.YYNEAGYEKQRGTQLGN.EN.....SRVYNEMAIIKIAQSTV..
Hs_UBE2Z_14       108    PAQS.ISSVLISIQSLM.TENP.YHNEPGFEQERHPG.....D.....SKNYNECIRHETIRVAV..
Hs_UBE2L6_15       96    PCTK.TCQVLEALNV.LVNRP..NIREPLRMDLADLLTQ...N.....PELFRKNAEEFTLRFGVD.
Hs_UBE2L3_15       97    PATK.TDQVIQSLIA.LVNDP..QPEHPLRADLAEEYSK...D.....RKKFCKNAEEFTKKYGE..
Ce_Ubc18_15        97    PATK.TEQVMMALLS.LINEP..EPSHPIRADVAEEFQK...D.....HKKFMKTAEEHTRKHAE..
Dm_CG17030_15     102    PTTR.IDQVLQVLLA.TINDP..QPENAWHIEMAGEYRN...D.....PVRFFKMADAWVQKYSE..
Ce_Ubc24_15       103    PETT.MEDVLLNLIV.LLNEP..DLSRPVNIDAAHDYIH...N.....KVEFVKKSTELAKKW....
Dm_UbcD84_15       97    PTTR.TEQVLQALVA.IVHNP..EPEHPLRSDLAEEFVR...E.....HKKFMKTAEEFTKKNAE..
```

Figure S2. Proteins of the E2 superfamily that only conserve the E2 fold but do not conserve the catalytic cysteine do not show a conserved CES/D site. An extract of the multiple sequence alignment of UEVs E2 families (family 10 and 16) with Ubc2 (Rad6) of family 2 (a serine at the CES/D site in bold) and Ubc1 of family 1 (a aspartate at the CES/D site in bold) as a reference.

Sc_Ubc2_2	-----A**SP**ANVEAATLFKDHKSQYVKRVKETVE
Sc_Ubc1_1	-----N**DP**QDAEVAQHYLRDRESFNKTAALWTR
Hs_UBE2V1_10	---LMMSKENMKLPQ--PPEGQCYSN-------
Hs_UBE2V2_10	---LMMSKENMKLPQ--PPEGQTYNN-------
Mm_UBE2V1_10	---LMMSKENMKLPQ--PPEGQCYSN-------
Mm_UBE2V2_10	---LMMSKENMKLPQ--PPEGQTYNN-------
Dm_Uev1A_10	---IMTMKENLKLAQ--PPEGSCF---------
Ce_Uev1_10	---MMMAKENLKLQQ--PAEGAMF---------
Sc_MMS2_10	---EMATPANKKLRQ--PKEGETF---------
Sp_MMS2_10	---EMASSSNRKLPQ--PPEGSTFF--------
At_Uev1C_10	---EMAASHNRKLVQ--PPEG-TFF--------
Hs_AKTIP_16	VVDSVKVCTARLFDQ--PKIEDPYAISFSPWNP
Mm_AKTIP_16	VVDSVKVCTARLFDQ--PKIEDPYAISFSPWNP
Dm_AKTIP2_16	VQEQAILSRNLIYDR--PPTEDPHYIIVEPYCA
Dm_AKTIP1_16	VQENIKESKEHIFDT--PPTEDPHYIVFEKFQQ
Ce_Ubc19_16	AKECVEASRSMVYDELAEQEHDPNGIRLLPWDA
At_ELCL_16	SAREVETEGLLSLQSDLKRREEEINNGFKEMVI

Figure S3. Multiple sequence alignment by ClustalW to define the corresponding residues of the different E2 enzymes for PCA analysis.

```
hHR6A           MSTPARRRLMRDFKRLQEDPPAGVSGAPSEN-----NIMVWNAVIFGPEGTPFEDGTFKL 55
hHR6B           MSTPARRRLMRDFKRLQEDPPVGVSGAPSEN-----NIMQWNAVIFGPEGTPFEDGTFKL 55
RAD6            -STPARRRLMRDFKRMKEDAPPGVSASPLPD-----NVMVWNAMIIGPADTPYEDGTFRL 54
UBE2I           MSGIALSRLAQERKAWRKDHPFGFVAVPTKNPDGTMNLMNWECAIPGKKGTPWEGGLFKL 60
UBC1            ---SRAKRIMKEIQAVKDDPAAHITLEFVSES----DIHHLKGTFLGPPGTPYEGGKFVV 53
                       *: :: :  :.* .  .      :     ::   :  : *  .**:*.* * :

hHR6A           TIEFTEEYPNKPPTVRFVSKMFHPNVY-ADGSICLDILQN--RWSPTYDVSSILTSIQSL 112
hHR6B           VIEFSEEYPNKPPTVRFLSKMFHPNVY-ADGSICLDILQN--RWSPTYDVSSILTSIQSL 112
RAD6            LLEFDEEYPNKPPHVKFLSEMFHPNVY-ANGEICLDILQN--RWTPTYDVASILTSIQSL 111
UBE2I           RMLFKDDYPSSPPKCKFEPPLFHPNVY-PSGTVCLSILEEDKDWRPAITIKQILLGIQEL 119
UBC1            DIEVPMEYPFKPPKMQFDTKVYHPNISSVTGAICLDILKN--AWSPVITLKSALISLQAL 111
                 : .  :** .**  :* . ::***:    * :**.**::   * *.  : . * .:* *

hHR6A           LDEPNPNSPANSQAAQLYQENKREYEKRVSAIVEQSWRDC-- 152
hHR6B           LDEPNPNSPANSQAAQLYQENKREYEKRVSAIVEQSWNDS-- 152
RAD6            FNDPNPASPANVEAATLFKDHKSQYVKRVKETVEKSWEDDMD 153
UBE2I           LNEPNIQDPAQAEAYTIYCQNRVEYEKRVRAQAKKFAPS--- 158
UBC1            LQSPEPNDPQDAEVAQHYLRDRESFNKTAALWTRLYAS---- 149
                ::.*:  .* : :.   :  .: .: * .   ..
```

~

Figure S4. Snapshots from the MD simulations of the wild type, mutant and phosphorylated Ube2I-RanGAP1 complexes. In all the structures, the residue at the 127 site (CES/D site) is shown on the left, the catalytic cysteine in yellow, and the Glu98 on the right. RanGAP1 and the E2 enzyme are shown as grey and pink cartoons, respectively. The Ube2I$_{D127A}$-RanGAP1 is not reported here since it provides results similar to the Ube2I$_{D127T}$-RanGAP1 simulation.

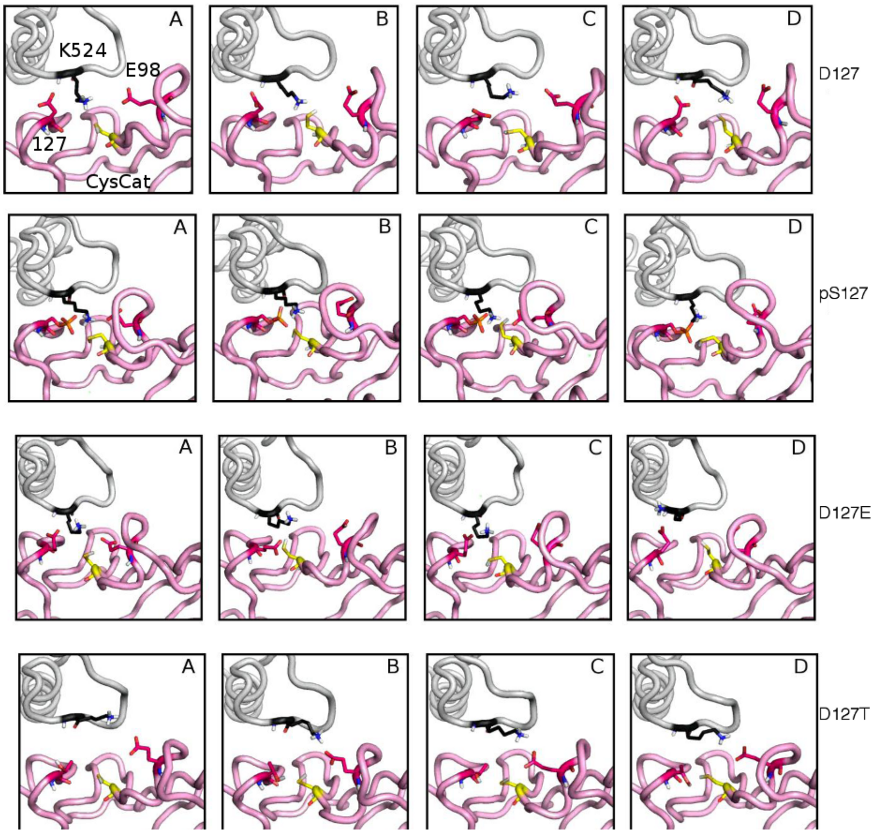

Figure S5. Main-chain RMSD over the simulation time of each hHR6A MD replicates reported in Table S2. A) wild-type hHR6A B) S120A hHR6A C) S120D hHR6A D) pS120 hHR6A E) S120E hHR6A F) S120T hHR6A

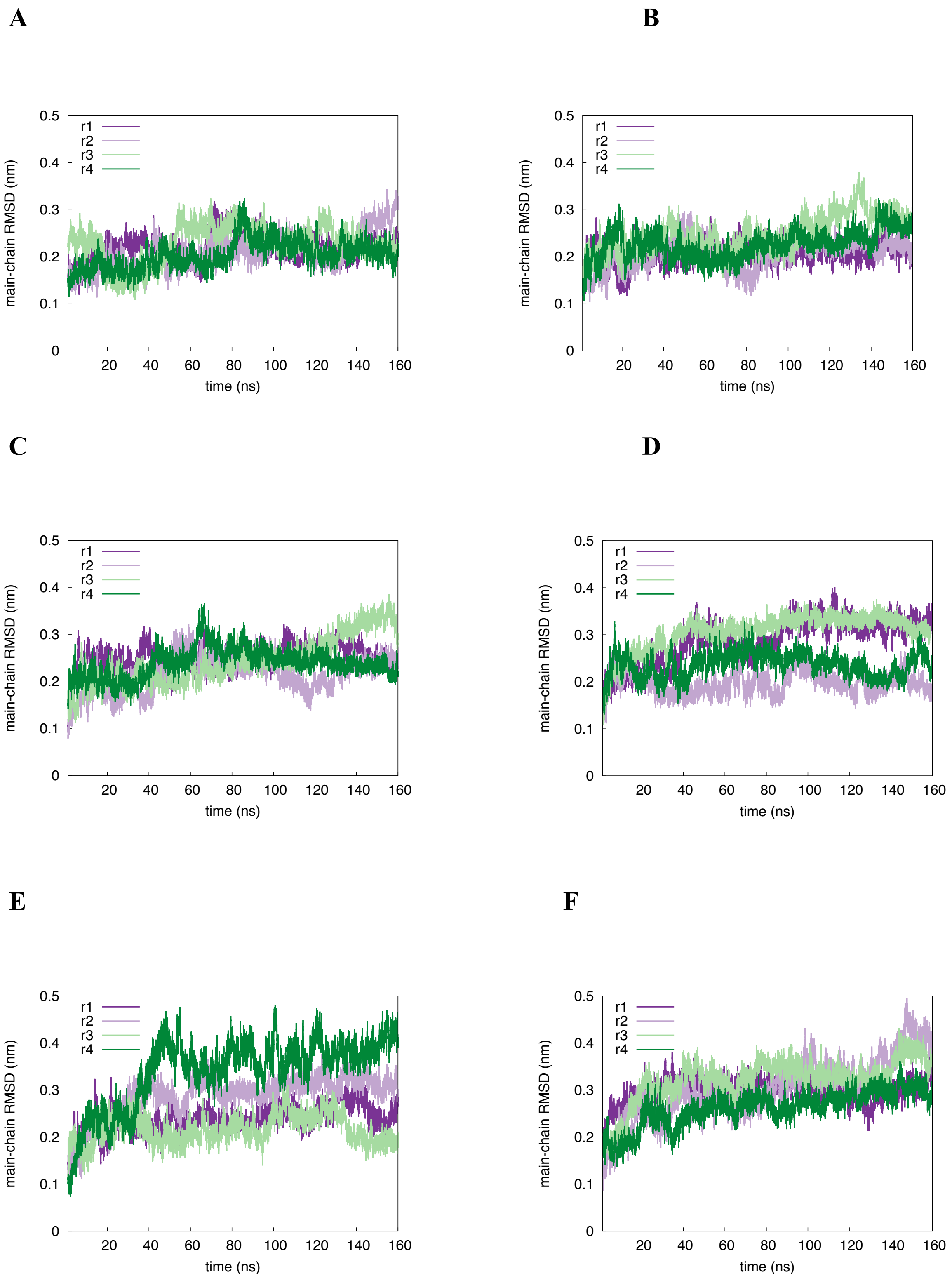

Figure S6. Main-chain RMSD over the simulation time of each Rad6, hHR6B, Ubc1 and Ube2I MD replicates reported in Table S2. A) wild-type Rad6 B) S120A Rad6 C) S120D Rad6 D) pS120 Rad6 E) hHR6B F) Ubc1 G) Ube2I

A

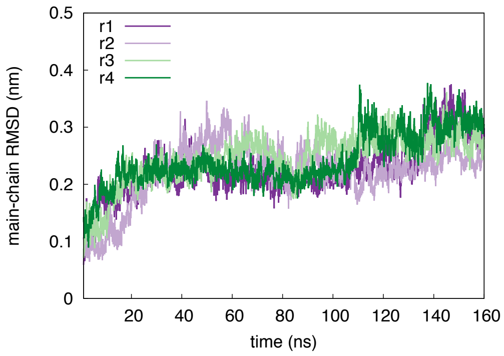

B

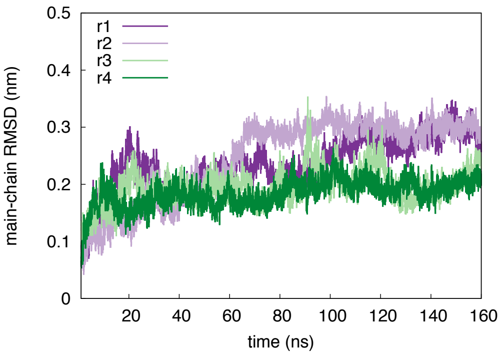

C

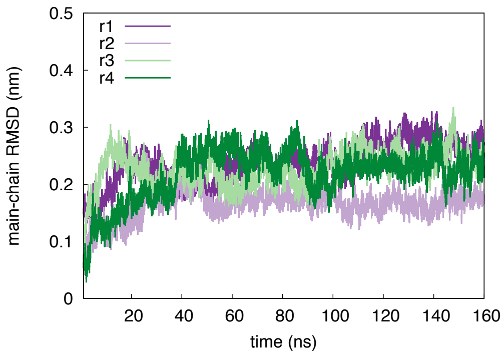

D

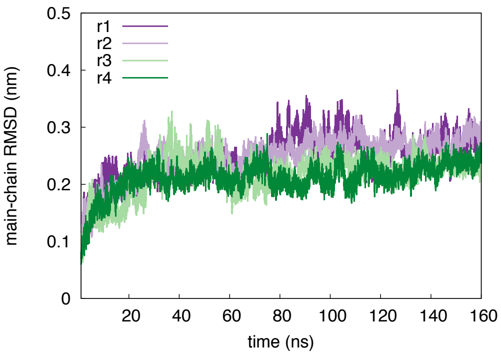

E

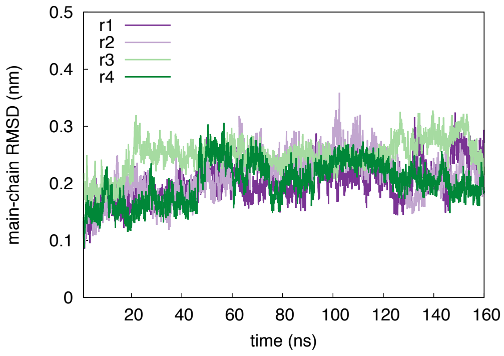

F

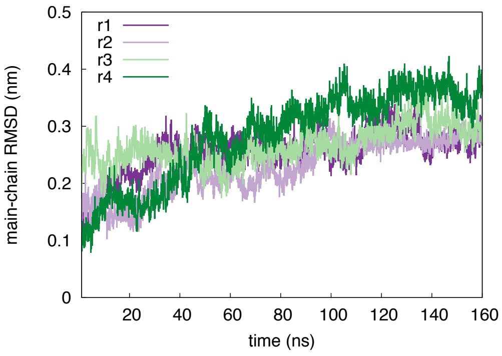

G

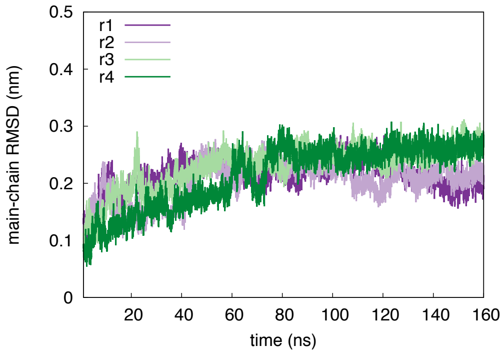

r1
r2
r3
r4
main-chain RMSD (nm)
0.5
0.4
0.3
0.2
0.1
0
20
40
60
80
100
120
140
160
time (ns)

Figure S7. Main-chain RMSD over the simulation time of each Ube2I-RanGap1 MD replicates reported in Table S2. A) wild-type complex B) D127A complex C) D127S complex D) pS127 complex E) D127E complex F) D127T complex. We should notice that in these simulations most of the complex residues have been restrained at their atomic positions using and harmonical potential (see Methods).

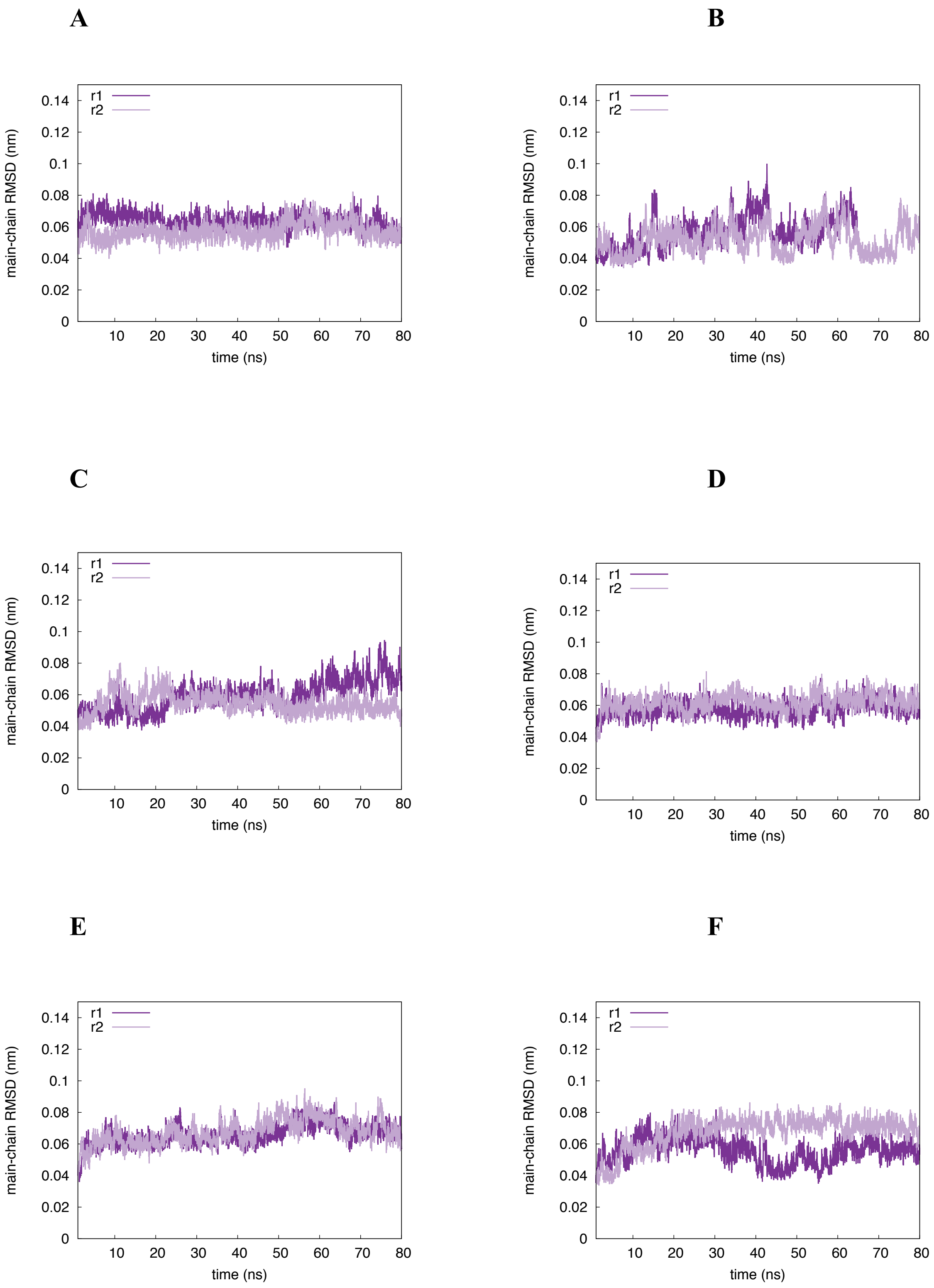

Supplement: Supplementary Information [file srep14849-s1.pdf]
